# Complement-amplified thrombotic microangiopathy in systemic lupus erythematosus and lupus nephritis: a systematic review of 78 reported patients

**DOI:** 10.3389/fimmu.2026.1940769

**Published:** 2026-09-30

**Authors:** Sheng-Guang Li, Ruohan Yu, Yanfeng Zhang, Lina Zhang, Jing Zhang, Yadan Zou, Ji Li, Ting Long, Xujie You

**Affiliations:** Department of Rheumatology and Immunology, Peking University International Hospital, Beijing, China

**Keywords:** atypical hemolytic uremic syndrome, complement, eculizumab, lupus nephritis, systematic review, systemic lupus erythematosus, thrombotic microangiopathy

## Abstract

**Background:**

Thrombotic microangiopathy (TMA) complicating systemic lupus erythematosus (SLE) and lupus nephritis (LN) spans immune thrombotic thrombocytopenic purpura, anti-phospholipid-associated microangiopathy, secondary endothelial injury, and complement-amplified/atypical hemolytic uremic syndrome (aHUS)-like phenotypes. The boundaries remain uncertain, and treatment evidence is dominated by selectively published cases.

**Methods:**

In this PROSPERO-registered systematic review (CRD420261409684), we searched PubMed, Embase, and Web of Science through to 17 March 2026, verified the source identity and eligibility, and performed a PubMed-only update through to 14 August 2026. We reconstructed patient-level reports by phenotype, competing mechanisms, complement-related evidence, treatment, and outcome. We included citable published abstracts when patient-level data were extractable but analyzed them as a lower-information stratum rather than as equivalent to full texts. Analyses were descriptive. We did not include China National Knowledge Infrastructure (CNKI) because no verifiable search record was available. One reviewer performed the screening, extraction, and appraisal. We report both as protocol deviations.

**Results:**

We included 56 sources describing 78 patients: in total, 39 full-text sources/61 patients and 17 published abstract sources/17 patients. There were 56 female patients (71.8%), and the median age was 24.5 years. Source-adjudicated microangiopathic hemolysis, thrombocytopenia, acute kidney injury/renal failure, strict TMA identified on kidney biopsy, and dialysis/renal replacement therapy (RRT) were present in 71 (91.0%), 74 (94.9%), 67 (85.9%), 42 (53.8%), and 46 (59.0%), respectively. Low C3/C4 levels were reported in 55 (70.5%), strict T2 complement evidence in 1 (1.3%), and index event terminal/tissue activation in 6 (7.7%). Complement inhibitors were reported in 60 (76.9%) and renal and hematologic improvements in 49 (62.8%) and 65 (83.3%), respectively. Certainty was very low, and treatment comparisons were not estimable.

**Conclusions:**

SLE/LN-associated TMA is heterogeneous, and complement may act as a driver, an amplifier, or an epiphenomenon. Case-based evidence supports rigorous mechanistic adjudication and biomarker-rich registries, but cannot establish incidence, prognosis, comparative efficacy, or universal treatment duration.

**Systematic review registration:**

https://www.crd.york.ac.uk/PROSPERO/view/CRD420261409684, identifier CRD420261409684.

## 1 Introduction

Systemic lupus erythematosus (SLE) is a prototypical immune complex autoimmune disease in which autoantibodies, complement activation, inflammatory cells, endothelial perturbation, and thrombosis may converge in the same patient. Lupus nephritis (LN) remains a major determinant of long-term morbidity and kidney survival. Contemporary LN classification and treatment recommendations are organized predominantly around glomerular immune complex injury, histologic activity and chronicity, proteinuria, and kidney function (1–4). The renal vascular compartment, however, carries additional diagnostic and prognostic information. In a large biopsy cohort, vascular immune deposits, arteriosclerosis, necrotizing vasculopathy, vasculitis, and thrombotic microangiopathy (TMA) were common enough to warrant systematic reporting, and the inclusion of vascular lesions improved renal-outcome prediction beyond glomerular class alone (5).

TMA is both a clinicopathological syndrome and a final common pattern of microvascular endothelial injury. Systemic clinical TMA is commonly recognized through microangiopathic hemolytic anemia, thrombocytopenia, and acute organ injury. Renal-limited TMA may instead be detected on kidney biopsy through endothelial swelling, luminal narrowing, subendothelial expansion, mesangiolysis, fibrin- or platelet-rich thrombi, or chronic double contours and fibrous intimal changes, even when the full hematologic triad is absent. Across LN biopsy cohorts, renal TMA has generally been associated with more severe kidney dysfunction and adverse outcomes, although the magnitude and independence of this association have varied after adjustment for baseline creatinine, proteinuria, and chronic damage (6–10). These differences argue against treating every vascular lesion, or every reported “aHUS” (atypical hemolytic uremic syndrome) diagnosis, as a biologically uniform entity.

The diagnostic problem is particularly difficult in SLE because several TMA mechanisms may coexist. Immune thrombotic thrombocytopenic purpura (TTP), anti-phospholipid syndrome (APS) or catastrophic APS (CAPS), pregnancy- or postpartum-associated TMA, malignant hypertension, disseminated intravascular coagulation (DIC), infection, drug toxicity, transplantation, and severe immune complex LN can each produce overlapping hematologic, renal, neurologic, and vascular findings. Pregnancy further compresses the differential among preeclampsia/HELLP (hemolysis, elevated liver enzymes, and low platelet count) syndrome, TTP, and complement-mediated TMA (11); malignant hypertension can itself produce microangiopathic hemolysis and renal failure (12); and consumptive coagulopathy or drug exposure may generate additional mimics (13, 14). SLE is therefore not proof that TMA is lupus-driven, while identification of another trigger does not necessarily exclude complement amplification or a clinically meaningful overlap.

Complement biology provides a plausible bridge between immune complex LN and microvascular injury, but evidentiary levels must be distinguished. In primary complement-mediated aHUS, genetic or acquired failure of complement regulation permits uncontrolled activation on susceptible endothelial surfaces; in other kidney diseases, complement may be secondary to tissue injury, but still amplifies inflammation and thrombosis (15, 16). Low C3 or C4 levels are common in active SLE and have limited specificity for complement-mediated TMA. Complement gene findings require variant-level interpretation because the panel composition, population frequency data, and classification standards have evolved (17, 18). Anti-factor H antibodies represent a distinct acquired mechanism (19), whereas soluble or tissue terminal pathway activation provides more proximal evidence of complement engagement. Even these findings do not, in isolation, distinguish a primary complement disorder from a secondary activation.

SLE-associated immunothrombosis offers additional mechanistic coherence. Impaired degradation of neutrophil extracellular traps (NETs) has been associated with renal involvement in SLE (20), while oxidized mitochondrial DNA within NETs can activate STING-dependent type I interferon pathways and intensify lupus-like inflammation (21). Lupus low-density granulocytes can damage endothelial cells and impair endothelial repair (22). Activated platelets can enhance plasmacytoid dendritic cell interferon-α production through CD154–CD40 signaling (23). Complement can also intersect directly with coagulation: in anti-phospholipid–antibody experiments, C5a receptor signaling induced neutrophil tissue factor and increased procoagulant activity (24). These observations support a network linking immune complexes, NETs, platelets, complement, endothelium, and coagulation, but do not demonstrate that this network caused TMA in any individual case.

The clinical literature remains dominated by case reports, small series, letters, and published abstracts spanning more than two decades of changing terminology, diagnostics, and treatment access. Authors have used “HUS,” “aHUS,” “complement-mediated TMA,” “TTP-like microangiopathy,” and “renal TMA” with inconsistent criteria. Some cases contain complement genetics, anti-factor H testing, or terminal pathway evidence, while others rely on hypocomplementemia, biopsy morphology, treatment refractoriness, or improvement after complement inhibition. A response to eculizumab is clinically informative, but cannot retrospectively validate the diagnosis when glucocorticoids, immunosuppressants, plasma exchange (PLEX), dialysis, and supportive care are administered concurrently. We therefore use complement-amplified TMA as a mechanistic hypothesis and an aHUS-like phenotype as a descriptive label, not as proof of primary inherited aHUS (**Figure 1**).

**Figure 1:**
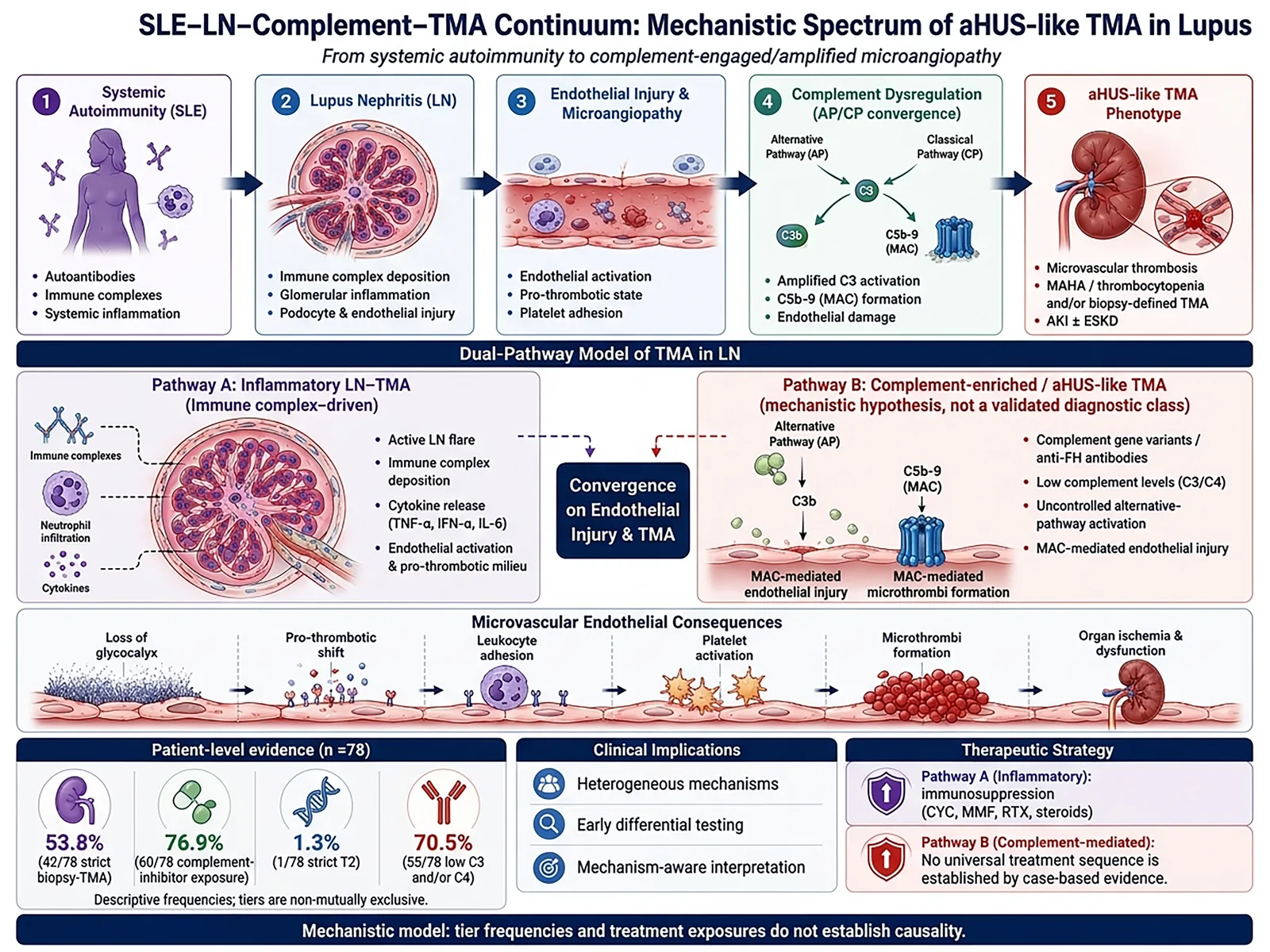
Systemic lupus erythematosus (SLE)–lupus nephritis (LN)–complement–thrombotic microangiopathy (TMA) continuum: mechanistic spectrum of an atypical hemolytic uremic syndrome (aHUS)-like TMA phenotype in lupus. Systemic autoimmunity and immune complex nephritis may converge on endothelial injury and a complement-engaged or complement-amplified phenotype. The *lower panel* reports selected descriptors from the source-adjudicated 78-patient dataset. These frequencies do not establish causality, a uniform diagnostic category, or treatment efficacy.

We undertook a systematic review of patient-level case-based evidence to characterize this spectrum without collapsing distinct constructs. The objectives were to distinguish systemic clinical TMA, renal-limited or biopsy-defined TMA, and author-labeled aHUS; grade the mechanistic proximity of reported complement-related evidence; preserve competing and overlapping mechanisms; reconstruct treatment sequences; harmonize the renal and hematologic outcomes transparently; evaluate the certainty and reporting limitations of the evidence; and define the minimum evidence needed for future prospective studies.

## 2 Methods

### 2.1 Protocol, registration, chronology, and deviations

We report this review in accordance with the Preferred Reporting Items for Systematic Reviews and Meta-Analyses (PRISMA) 2020 statement and PRISMA-S (25, 26). We registered the protocol in PROSPERO as CRD420261409684 under the title “Clinical characteristics, treatment response, and renal outcomes of systemic lupus erythematosus/lupus nephritis-associated atypical hemolytic uremic syndrome phenotype or complement-mediated thrombotic microangiopathy: a systematic review of case-based evidence” (27). The registered start and anticipated completion dates were 29 May and 30 June 2026. The database exports were generated on 17 March 2026, before the registered start date. Accordingly, registration was not prospective for the identification stage, and we did not perform a separate May/June search.

We report three material protocol deviations. First, although PROSPERO listed the China National Knowledge Infrastructure (CNKI), we found no verifiable CNKI export, search history, or screening log; therefore, our reproducible search comprised PubMed, Embase, and Web of Science, and we did not include CNKI. We describe the resulting database/language bias risk in the source flow results, **Supplementary Table S1**, and limitations. Second, only one reviewer performed the screening, extraction, and JBI appraisal rather than two independent reviewers, as registered. We verified the source identity, eligibility, linkage, complement, and treatment fields, along with patient-keyed outcomes against the available primary source text with computational assistance; this verification did not constitute dual-independent human screening or dual-human extraction. Third, we performed the registered GRADE-informed assessment described in *Section 2.9*.

### 2.2 Eligibility criteria

Eligible publications contained extractable, individual-level data for patients of any age or sex with SLE or LN and at least one of three routes: a systemic clinical TMA phenotype; a renal biopsy TMA or strongly compatible renal microvascular injury; or an author-assigned diagnosis of HUS, aHUS, complement-mediated TMA, or TTP-like microangiopathy. Case reports, case series, letters, cohorts with separable patient data, and publicly disseminated, citable published abstracts with sufficient clinical, treatment, and outcome information were eligible. Abstract-level records were eligible, but constituted a lower-information stratum and were never treated as evidentially equivalent to a full report. Published abstracts were not equated with unpublished material: unpublished manuscripts, private reports, personal communications, and non-public datasets were ineligible. Aggregate-only cohorts, reviews without unique patient data, animal or *in vitro* studies, reports without an interpretable SLE/LN association, and duplicate descriptions were excluded. Reports attributable solely to another primary cause were excluded, but cases with plausible overlapping mechanisms were retained for multi-label adjudication.

Classic immune TTP, APS/CAPS, pregnancy-associated TMA, malignant hypertension, infection/DIC, drug-induced TMA, transplantation-associated TMA, and refractory active LN were not assumed to be absent because the authors used an aHUS label. Available evidence for these alternatives or co-drivers was extracted and incorporated into adjudication. Published abstracts were retained as a separate lower-information stratum, checked for overlap or a later full report, and examined in a full text-only sensitivity analysis; their inclusion did not upgrade their information content or certainty.

### 2.3 Information sources and search strategy

We searched PubMed/MEDLINE, Embase, and the Web of Science Core Collection through to 17 March 2026 without a user-applied year restriction using the coverage available through each platform. We combined controlled vocabulary and free-text terms for SLE/LN with TMA, HUS/aHUS, complement-mediated microangiopathy, hemolysis, and renal microvascular injury and adapted the syntax to each platform. We classified the reference list and author-known records under “other methods” rather than the database search lane. Although PROSPERO listed CNKI, we could not verify a CNKI search in the available records and therefore did not include it. We treated this as a protocol deviation, not a negative search.

We checked the source identity, eligibility, attachment accuracy, citation identity, omissions, and overlap. We also updated PubMed from 18 March to 14 August 2026. We did not rerun Embase or Web of Science to the August date and did not include CNKI; therefore, the database searches did not share a common end date. **Supplementary Tables S1–S4** provide the search strategies, source decisions, crosswalk, and protocol deviations.

### 2.4 Study selection, source linkage, and overlap control

After deduplication, the records underwent title/abstract screening, followed by a full-record eligibility assessment. Potential duplicate or overlapping patients were compared using authorship, institution, country, demographic characteristics, chronology, pregnancy status, pathology, treatment sequence, genetic findings, and outcome. When reports described the same patient, the most complete source was considered the canonical citation, and only clearly non-duplicative supplementary information was carried forward. Multi-patient series contributed separate patient rows, but remained source-clustered.

Our source integrity checks identified Fukunaga 2026 in the PubMed export as eligible. Khan 2020 and Theodoru 2023 were also eligible after verification of their published abstracts showed that the locally linked PDFs were unrelated. We identified three eligible pre-cutoff reports outside the database exports (Stirnkorb 2026, Baltaci 2026, and Kaneda 2020) and classified them under other methods/source integrity checking. We excluded Pickering 2015 and Patel 2025 at the report-level assessment: Pickering explicitly described non-TMA glomerular lesions, whereas Patel concerned allograft C3 glomerulonephritis without current clinical or biopsy TMA (28, 47). **Supplementary Table S3** provides the citation-level decisions.

The diagnostic framework was deliberately multi-label (**Figure 2**). Panel A shows non-directional, non-mutually exclusive adjudication domains. Panel B quantifies four reproducibly coded contextual/evidence domains: strict complement-related evidence (C), APS/CAPS wording (A), malignant/severe hypertension (H), and pregnancy/postpartum context (P). Across these four domains, out of 78 patients, 11 had none coded, 46 had one, 19 had two, and 2 had three; therefore, 21/78 had at least two coded domains. This selected four-domain overlap does not imply complete ascertainment of infection, active LN activity, or other exposure-related mechanisms. Treatment exposure and response are downstream observations and were not used to define the mechanism.

**Figure 2:**
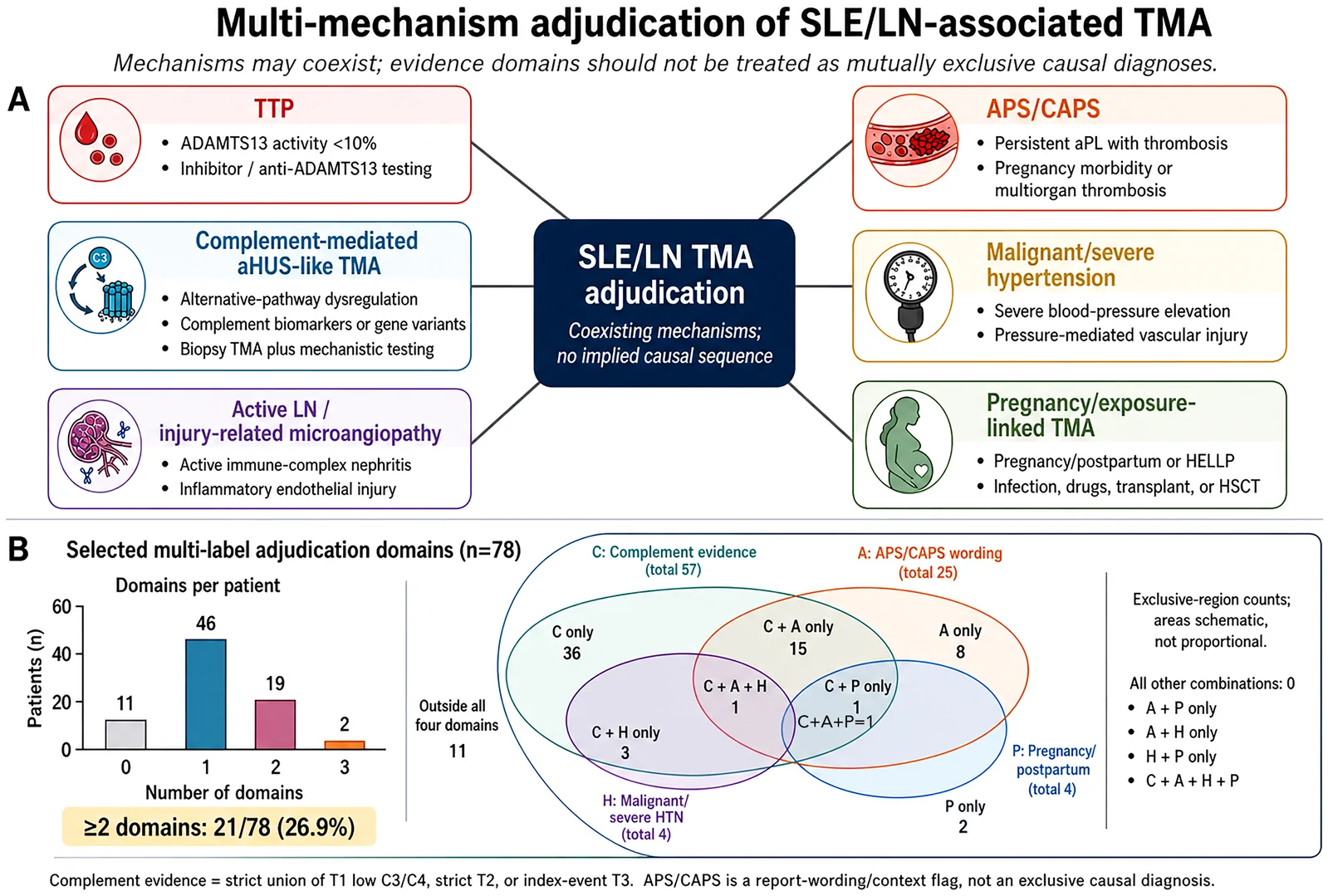
Diagnostic adjudication of systemic lupus erythematosus (SLE)/lupus nephritis (LN) with a thrombotic microangiopathy (TMA) phenotype. **(A)** The observed systemic or kidney-predominant TMA phenotype is assessed in parallel across six non-directional, non-mutually exclusive domains. The test timing, treatment timing, negative findings, and unreported domains should be documented before assigning a complement-mediated phenotype. Response to complement inhibition is contextual, confounded evidence, and is not a diagnostic criterion. **(B)** Four reproducibly coded domains are shown as an exclusive region overlap map: strict complement-related evidence **(C)**, anti-phospholipid syndrome (APS)/catastrophic APS (CAPS) wording (*A*), malignant/severe hypertension (*H*), and pregnancy/postpartum context (*P*). The exclusive region counts, including C + A + P = 1, sum up to 78. The accompanying distribution is as follows: zero domain, 11; one domain, 46; two domains, 19; and three domains, 2 (at least two domains, 21/78). Infection, active LN activity, and exposure-related mechanisms beyond pregnancy/postpartum were not included in **(B)** because ascertainment was inconsistent.

### 2.5 Data extraction and management of missing information

The patient-level dataset captured source type and year; country; age and sex; SLE/LN status; pregnancy or postpartum context; kidney pathology and LN class; thrombocytopenia, hemolysis, kidney injury, blood pressure, RRT, and extrarenal involvement; ADAMTS13/TTP assessment; anti-phospholipid antibodies or APS/CAPS; infection, coagulation, drug, and transplantation contexts; complement concentrations, functional assays, genetics, anti-factor H antibodies, terminal pathway markers, and tissue staining; and immunosuppression, plasma therapy, anti-thrombotic treatment, complement inhibitors, treatment sequence, follow-up, harms, and outcomes. Missing values were not imputed. “Not reported,” “not tested,” “unclear,” and an explicitly negative result remained distinct states.

One source reported age only as “in the 30s.” We used a prespecified midpoint of 35 years for the primary age summary and excluded that value in a sensitivity check. One included source contained an internal creatinine transcription discrepancy. We used the primary source value in the patient-level matrix (**Supplementary Table S5**). We linked the clinical source text and the structured dataset by stable patient and source keys.

### 2.6 Phenotype adjudication and complement-evidence tiers

Systemic clinical TMA was coded when the source documented microangiopathic hemolytic anemia, thrombocytopenia, and acute organ injury. Individual components were preserved, and thus, incomplete triads were not silently recoded as negative. Renal-limited or biopsy TMA was coded separately. A strict biopsy definition required explicit TMA terminology in the pathology interpretation, while a broader rule accepted strongly compatible endothelial or microvascular descriptions. Strict RRT required explicit dialysis/RRT wording, while a broader rule incorporated unambiguous narrative descriptions.

Author-labeled aHUS was recorded verbatim as source attribution, but not accepted as confirmation of primary complement-mediated disease. Each case was reviewed for severe ADAMTS13 deficiency/TTP, APS/CAPS, pregnancy-related TMA, malignant hypertension, DIC/sepsis, drug-induced TMA, transplantation, and severe active LN. Pregnancy, malignant hypertension, DIC, and drug exposure signals were interpreted using established constructs rather than labels alone (11–14).

Complement findings were coded in three non-mutually exclusive tiers, and a patient meeting more than one tier was counted in every applicable tier. Tier 1 (T1) required explicitly low C3 and/or C4 levels. Unspecified “hypocomplementemia” was retained separately and not promoted to T1. Strict tier 2 (T2) required a literal pathogenic/likely pathogenic complement finding or positive anti-factor H antibody. The extended T2 sensitivity definition additionally accepted a source-described deleterious or author-causal complement finding without a formal pathogenic/likely pathogenic classification. Variants of uncertain significance (VUS), uncertain copy number changes, and unclassified findings were reported separately and never promoted to strict T2 (17–19). Tier 3 (T3) required direct terminal pathway or tissue complement activation documented at the index event, and timing-uncertain findings were retained, but not counted. Clinical response to complement inhibition was excluded from the tiers to avoid circular diagnosis. Strict and extended unions and exact overlap distributions were calculated explicitly.

### 2.7 Treatment coding and *post-hoc* outcome harmonization

Treatments were coded as non-mutually exclusive, administered exposures, with any-time and index episode exposure separated. “Not reported” was not treated as “no.” Recorded categories included glucocorticoids, cyclophosphamide, mycophenolate mofetil, B-cell-directed agents, intravenous immunoglobulin, calcineurin inhibitors (CNIs), PLEX, anticoagulation and antiplatelet therapy, dialysis, transplantation, and complement inhibitors. Complement agents were recorded separately as eculizumab, ravulizumab, pegcetacoplan, and iptacopan/LNP023. Rituximab and obinutuzumab were recorded separately as B-cell-directed anti-CD20 therapies and were not classified as complement inhibitors.

For the patient-keyed outcome display, we assigned patients to one mutually exclusive index complement inhibitor class: no complement inhibitor; eculizumab only; or another complement sequence containing ravulizumab, pegcetacoplan, and/or iptacopan. These are treatment histories, not comparable interventions. We classified the renal status at the last reported follow-up as improved/recovered or dialysis-independent, persistent kidney function loss/dialysis dependence, death without renal recovery or explicit no recovery, or unclear. We classified hematologic status as improved, no response, or unclear and retained unclear outcomes in the denominator. We generated **Figure 3** from the 78-row frozen dataset.

**Figure 3:**
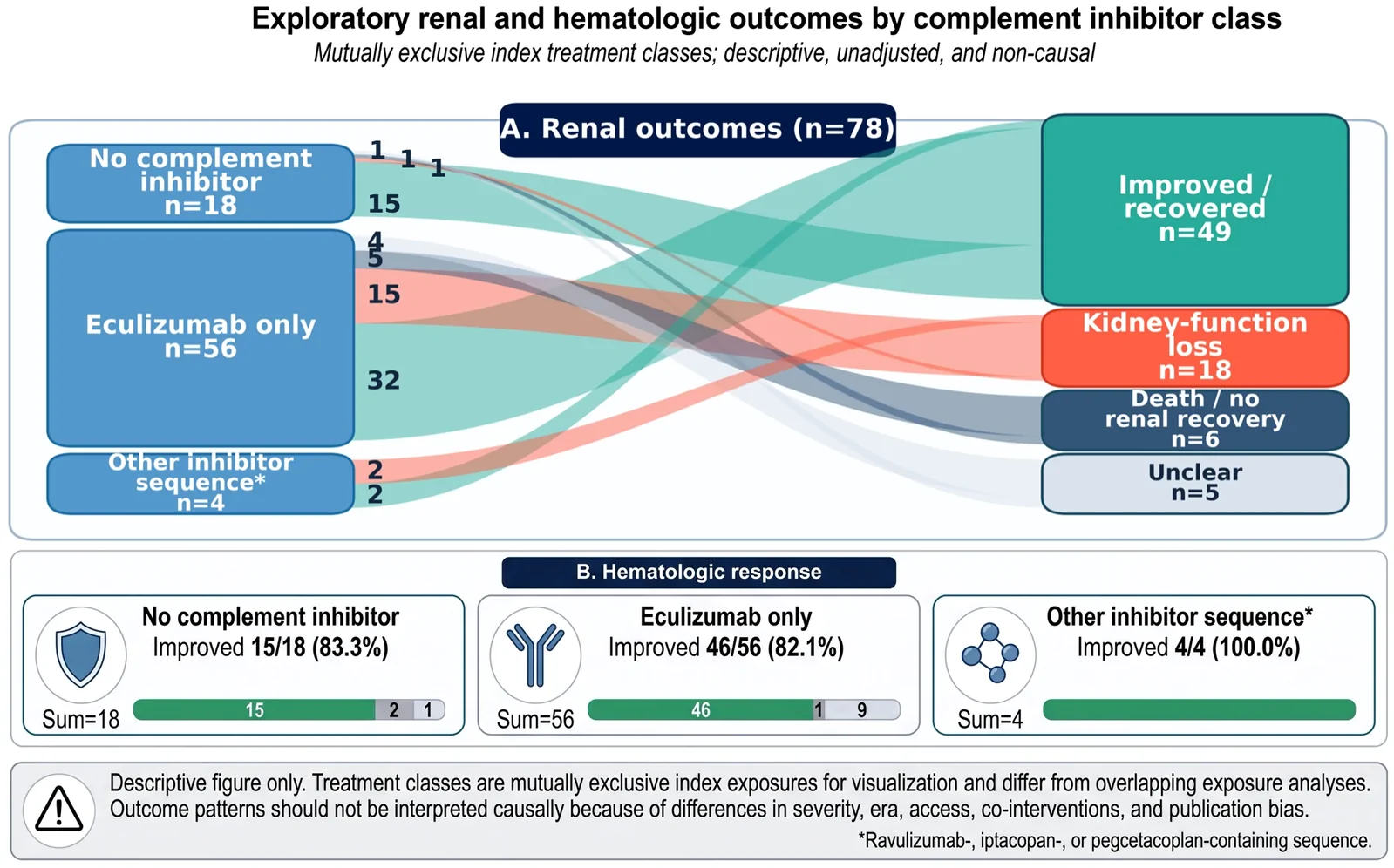
Exploratory renal and hematologic outcomes by index complement inhibitor class. **(A)** Sankey diagram displaying the renal outcomes among the 78 patients assigned to the following mutually exclusive index classes: no complement inhibitor (*n* = 18), eculizumab only (*n* = 56), or another complement inhibitor sequence (*n* = 4). Renal outcomes were improved/recovered (*n* = 49), kidney function loss (*n* = 18), death/no renal recovery (*n* = 6), or unclear (*n* = 5). **(B)** Hematologic improvement, no response, and unclear outcomes are shown separately. The display is descriptive and unadjusted. The severity, era, access, co-interventions, selective reporting, and confounding by indication preclude causal comparisons.

### 2.8 Reporting quality appraisal

We appraised full-text single-case reports with the JBI Case Reports checklist and multi-patient reports with the JBI Case Series checklist (29, 30). We assessed published abstracts separately for the reporting of demographics, clinical history, diagnostic evidence, treatment sequence, outcomes/follow-up, and harms. We used project-level “high” or “moderate” labels only to summarize reporting completeness. These labels are not official JBI grades, risk-of-bias scores, or certainty ratings. One reviewer conducted the JBI appraisal, and we did not perform duplicate human appraisal of every domain.

### 2.9 GRADE-informed certainty assessment

In accordance with the registered protocol, certainty was assessed narratively across clinically important outcome domains using GRADE concepts: study limitations/risk of bias, inconsistency, indirectness, imprecision, and publication bias (31). Because all the eligible patient-level evidence consisted of uncontrolled, selectively published cases or small series, the initial certainty level was low and was downgraded for multiple serious limitations. The assessment addresses the certainty of the descriptive findings among published cases, but does not transform those proportions into incidence, prognosis, or treatment effects. Where no credible comparator or denominator existed, comparative effects were classified as not estimable rather than being assigned spurious precision.

### 2.10 Statistical synthesis and sensitivity analyses

Categorical variables were summarized as counts over explicit denominators and percentages, and age was summarized using the median, interquartile range, and range. Patient-level percentages are descriptive because patients from the same source were clustered and the publication probability was unknown. Missing values remained unknown. No imputation, hypothesis testing, funnel plot, Egger test, or comparative effect estimate was performed. Such analyses are not valid for a small, heterogeneous collection of case reports without a sampling denominator.

We did not perform a meta-analysis because the evidence lacked comparator groups, standardized treatment timing, common outcome definitions, and uniform follow-up and was highly susceptible to publication and treatment selection bias. We followed the synthesis without meta-analysis (SWiM) principles (32). We prespecified the eligibility of sufficiently informative published abstracts. We treated the tiered abstract appraisal, full-text-only analysis, strict-*versus*-broad biopsy-TMA/RRT definitions, strict-*versus*-extended T2 analysis, exclusion of the author-affiliated Yu 2026 case, and the descriptive publication era and index treatment strata as *post-hoc* sensitivity or quality control analyses.

## 3 Results

### 3.1 Study selection and source integrity

Database searches yielded 598 records (PubMed, 118; Embase, 380; and Web of Science, 100). After removing 105 duplicates, we screened 493 records, excluded 451 at the title/abstract level, and assessed 42 reports. We excluded Patel 2025, leaving 41 reports in the database search lane.

Other methods/source integrity checks contributed 11 reports: seven manually identified reports that could not be linked to the 493-record database export, three eligible pre-cutoff reports identified through source verification, and the author-known Yu 2026 report. The PubMed-only update from 18 March to 14 August 2026 returned 19 records. We removed one duplicate of Yu, excluded 11 records at screening, assessed seven reports, excluded three aggregate reports without separable patient-level data, and included four. Across the three lanes, we included 41 from the database search, 11 from other methods, and four from the PubMed update (56 sources, 78 unique patients) (**Figure 4**; **Supplementary Table S3**) (33–46, 48–89).

**Figure 4:**
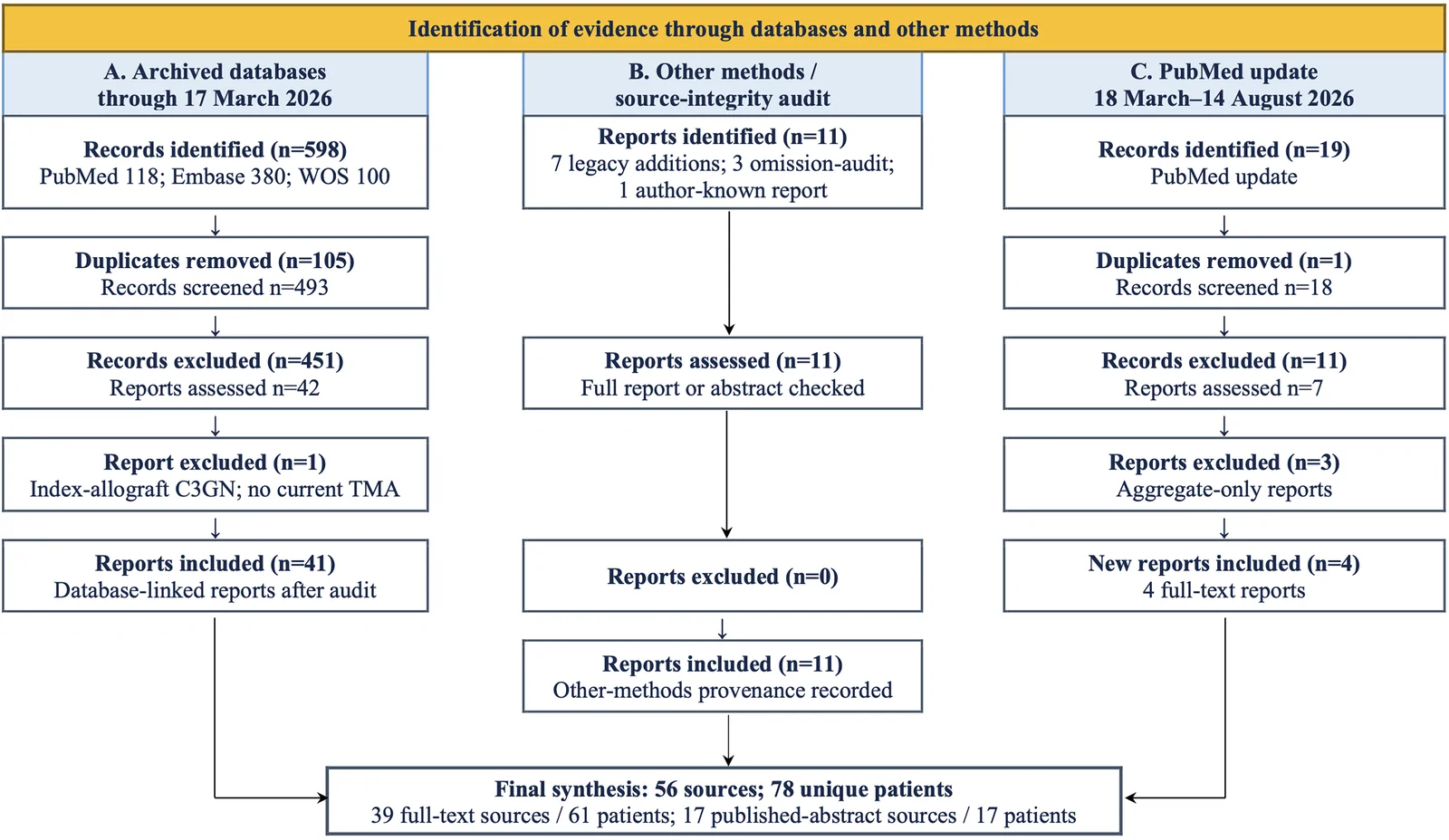
Preferred Reporting Items for Systematic Reviews and Meta-analysis (PRISMA) 2020 flow diagram. The three lanes show the database search records through 17 March 2026, the records identified through other methods/source integrity checking, and the PubMed update from 18 March to 14 August 2026. We included 56 sources and 78 unique patients: 39 full-text sources/61 patients and 17 published abstract sources/17 patients. The update lane was PubMed only. We did not include CNKI; this deviation from the registered search plan may introduce database and language bias. The current as construction incorrectly makes the possible bias sound like the reason for omitting CNKI.

There were 39 sources available as full text, which contributed 61 patients, while 17 publicly disseminated, citable published abstracts contributed one patient each. There were 16 conference abstracts and one peer-reviewed journal case available only at the abstract level. Five sources contributed more than one patient (11, 9, 3, 2, and 2 patients, respectively); the other 51 sources contributed one patient each. The source and patient denominators were therefore not interchangeable.

We identified Yu 2026 manually from an author-known accepted full text. The report was published on 20 July 2026 and was also retrieved in the PubMed update (89). Eight of the nine review authors co-authored that report. We did not use figure selection as an eligibility criterion and evaluated robustness with a leave-one-source-out sensitivity analysis. We disclose the authorship relationship in the Conflict of interest statement.

### 3.2 Demographic, renal, and TMA phenotype

Of the 78 patients, 56 were women (71.8%). The data regarding age at presentation were available for 76 patients (including one midpoint-coded “30s” report), with a median of 24.5 years (IQR = 20–35.25, range = 4–69). Of the patients, 11 (14.1%) were younger than 18 years old. These observations characterize selected reports, not population incidence.

Kidney pathology was available for 71 patients (91.0%), and class IV or mixed IV/V LN was reported for 38 patients (48.7%). Four-state source adjudication found microangiopathic hemolysis present in 71 patients (91.0%), thrombocytopenia in 74 (94.9%), and acute kidney injury/renal failure in 67 (85.9%). Strict renal biopsy TMA was present in 42 patients (53.8%), and strict dialysis/RRT was present in 46 (59.0%). “Absent,” “not reported,” and “unclear” were retained as distinct states. Neurologic involvement was reported in 13 patients (16.7%) and diffuse alveolar hemorrhage or other pulmonary involvement was reported in 9 (11.5%) (**Table 1**).

**Table 1:** Evidence base, demographics, phenotype, and reporting completeness.

Domain	Characteristic	Value
Evidence base	Included sources	56
Evidence base	Full-text sources/patients	39/61
Evidence base	Published abstract sources/patients	17/17
Demographics	Female sex	56/78 (71.8%)
Demographics	Age available	76/78; median = 24.5 years (IQR = 20–35.25, range = 4–69)
Demographics	Age <18 years	11/78 (14.1%)
Renal pathology	Class IV or mixed IV/V lupus nephritis	38/78 (48.7%)
Clinical phenotype	Thrombocytopenia present	74/78 (94.9%)
Clinical phenotype	MAHA/hemolysis present	71/78 (91.0%)
Clinical phenotype	AKI/renal failure present	67/78 (85.9%)
Clinical phenotype	Strict renal biopsy TMA present	42/78 (53.8%)
Clinical phenotype	Strict dialysis/RRT present	46/78 (59.0%)
Differential assessment	ADAMTS13/TTP assessment reported	55/78 (70.5%)
Context	APS/CAPS wording	25/78 (32.1%)
Context	Pregnancy/postpartum context	4/78 (5.1%)
Context	Diffuse alveolar hemorrhage/lung involvement	9/78 (11.5%)
Context	Neurologic involvement	13/78 (16.7%)
Context	Explicit malignant/severe hypertension wording	4/78 (5.1%)

Percentages use 78 patients, unless another denominator is shown. Age uses 76 available observations, including one midpoint-coded “30s” value. The source and patient denominators are displayed separately. The lupus nephritis (LN) class categories are not exhaustive.

MAHA, microangiopathic hemolytic anemia; AKI, acute kidney injury; TMA, thrombotic microangiopathy; RRT, renal replacement therapy; APS, anti-phospholipid syndrome; CAPS, catastrophic anti-phospholipid syndrome; IQR, interquartile range.

### 3.3 Differential-diagnostic and overlap signals

An ADAMTS13/TTP assessment was reported in 55 patients (70.5%), commonly as an activity result, inhibitor result, or explicit author statement. The source language suggesting TTP or severe reduction was retained as a flag, but no patient was upgraded to a confirmed case of immune TTP without an interpretable activity level below the contemporary threshold and in a context that was compatible. Missing pretreatment ADAMTS13 in 23 patients materially limited retrospective attribution.

The APS/CAPS wording was present in 25 patients (32.1%). The pregnancy/postpartum context was reported in four (5.1%) and explicit malignant or severe hypertension wording was reported in four (5.1%). In the four-domain overlap shown in **Figure 2B** (strict complement-related evidence, APS/CAPS, malignant/severe hypertension, and pregnancy/postpartum), out of 78 patients, 11 had no coded domain, 46 had one, 19 had two, and 2 had three; therefore, 21/78 had at least two coded domains. Infection, drugs, active LN, and other endothelial stresses occurred in individual narratives, but were incompletely standardized and were not included in this overlap count. These labels were not mutually exclusive, and their frequencies indicate reported co-context rather than single-cause diagnoses (**Table 2**).

**Table 2:** Domain-organized clinical, differential, and complement evidence.

Domain	Indicator	*n*/*N* (%)	Interpretation
Serology	T1: explicitly low C3 and/or C4 levels	55/78 (70.5%)	Common in active SLE; nonspecific for TMA mechanism
Genetic/acquired regulation	Strict T2: P/LP complement finding or positive anti-factor H	1/78 (1.3%)	Mechanistically closer; classification and timing remain important
Genetic/acquired regulation	Positive anti-factor H antibody	0/78 (0.0%)	No positive cases documented
Sensitivity definition	Extended T2	5/78 (6.4%)	Sensitivity analysis only; not equivalent to P/LP
Uncertain findings	VUS/uncertain CNV or unclassified complement finding	14/78 (17.9%)	Not counted as strict T2
Negative testing	At least one explicitly negative complement assay	28/78 (35.9%)	Panel scope and calendar era varied
Proximal activation	T3: index terminal-pathway or tissue complement activation	6/78 (7.7%)	Most proximal reported evidence; not proof of initiation
Composite	Strict union of T1, strict T2, or T3	57/78 (73.1%)	Non-mutually exclusive evidence map, not a diagnostic test
Composite	Extended union	60/78 (76.9%)	Sensitivity definition only

The domains are grouped as phenotype/pathology, differential diagnostic context, complement-related evidence, and clinical overlap. Tiers are non-mutually exclusive, and patients are counted in every applicable tier. “Strict union” refers to T1 and/or strict T2 and/or index T3; “extended union” refers to extended T2. A VUS or an uncertain finding is not a strict T2.

TMA, thrombotic microangiopathy; VUS, variants of uncertain significance; CNV, copy number variation; P/LP, pathogenic/likely pathogenic.

### 3.4 Complement evidence and exact overlap

Tier 1 (T1) evidence, defined as explicitly low C3 and/or C4 levels, was reported in 55 patients (70.5%). This common signal is compatible with active immune complex lupus and was not considered mechanism-specific. Strict T2 evidence was present in only one patient (1.3%), and no positive anti-factor H antibody was verified. Four additional records contained source-described deleterious or author-causal findings without formal pathogenic/likely pathogenic classification; thus, the extended T2 sensitivity category contained five patients (6.4%). There were 14 patients (17.9%) with a VUS, an uncertain copy number finding, or an otherwise unclassified complement finding, and 28 patients (35.9%) had at least one explicitly negative complement assay.

T3 evidence of terminal pathway or tissue activation at the index event was documented in six patients (7.7%), and one timing-uncertain finding was not counted. Under strict definitions, 57 patients (73.1%) had T1, strict T2, and/or T3 evidence. The exact strict overlap results were as follows: none, 21; T1 only, 50; T3 only, 2; T1+T3, 4; and T1+T2, 1. Under extended T2 definitions, the union was 60 (76.9%): none, 18; T1 only, 49; extended T2 only, 3; T3 only, 2; T1+T3, 4; and T1+extended T2, 2. **Table 2** reports the overlapping tiers rather than a mutually exclusive hierarchy.

### 3.5 Treatment landscape and publication era

Treatment was multimodal. PLEX was reported in 62 patients (79.5%). In one series, 11 patients relied on group-level exposure evidence, while 51 had patient-specific documentation. Exposures at any time included cyclophosphamide in 49 patients (62.8%), mycophenolate in 32 (41.0%), rituximab in 21 (26.9%), intravenous immunoglobulin in 13 (16.7%), and a CNI in 6 patients (7.7%). The index episode counts were 47, 25, 20, 13, and 3, respectively. Anticoagulation was reported in 16 patients (20.5%) and antiplatelet therapy in 4 (5.1%); 17 patients (21.8%) received at least one of these therapies. These are minimum documented exposures because unreported treatment was not recoded as non-use.

Any complement inhibitor was reported in 60 patients (76.9%). Complement agent exposures overlapped: eculizumab in 58 (74.4%), ravulizumab in 2 (2.6%), iptacopan in 1 (1.3%), and pegcetacoplan in 1 (1.3%). No obinutuzumab exposure was identified. Obinutuzumab is a B-cell-directed anti-CD20 therapy, not a complement inhibitor. The pegcetacoplan record was a published abstract and cannot establish efficacy. The mutually exclusive index classes comprised no inhibitor (*n* = 18), eculizumab only (*n* = 56), and another complement sequence (*n* = 4) (**Table 3**).

**Table 3:** Nested treatment exposures and reported outcomes.

Group/exposure	Patients/exposure	Index class outcomes
Complement inhibitor exposures (non-exclusive)		
Any complement inhibitor	60 (76.9%)	60 (76.9%)
Eculizumab	58 (74.4%)	56 in the eculizumab-only class
Ravulizumab	2 (2.6%)	Other sequence class
Iptacopan	1 (1.3%)	Other sequence class
Pegcetacoplan	1 (1.3%)	Other sequence class
Mutually exclusive index classes	Patients	Renal I/K/D/U; hematologic I/N/U
No complement inhibitor	18	15/1/1/1; 15/2/1
Eculizumab only	56	32/15/5/4; 46/1/9
Other complement sequence	4	2/2/0/0; 4/0/0
Other overlapping treatments (non-exclusive)	Any documented	Index episode
Plasma exchange (PLEX)	62 (79.5%)	62 (79.5%)
Cyclophosphamide	49 (62.8%)	47 (60.3%)
Mycophenolate mofetil	32 (41.0%)	25 (32.1%)
Rituximab	21 (26.9%)	20 (25.6%)
Intravenous immunoglobulin	13 (16.7%)	13 (16.7%)
Calcineurin inhibitor	6 (7.7%)	3 (3.8%)
Anti-coagulation	16 (20.5%)	16 (20.5%)
Anti-platelet therapy	4 (5.1%)	3 (3.8%)
Either antithrombotic class	17 (21.8%)	17 (21.8%)
B-cell therapy/anti-CD20: obinutuzumab	0	0; not a complement inhibitor

Complement agent and other treatment exposures overlap. The mutually exclusive index class block reports renal outcomes as improved/recovered (I), kidney function loss (K), death/no renal recovery (D), or unclear (U) and hematologic outcomes as improved (I), no response (N), or unclear (U). Counts describe reported administrations and outcomes, not comparative efficacy. “Not reported” does not mean “no”.

The publication era groups contained 13 patients in 2000–2014, 38 patients in 2015–2019, and 27 patients in 2020–2026. Complement inhibitor exposure was reported in 3/13 (23.1%), 35/38 (92.1%), and 22/27 patients (81.5%), while PLEX was reported in 10/13 (76.9%), 33/38 (86.8%), and 19/27 patients (70.4%) (**Figure 5**). This pattern reflects reported cases, evolving terminology, access, and selection—not population uptake or comparative effectiveness. **Figure 6** places stabilization, urgent adjudication and alternative-specific care, and LN-directed therapy before or alongside selected complement inhibition. It is not a first-line efficacy algorithm.

**Figure 5:**
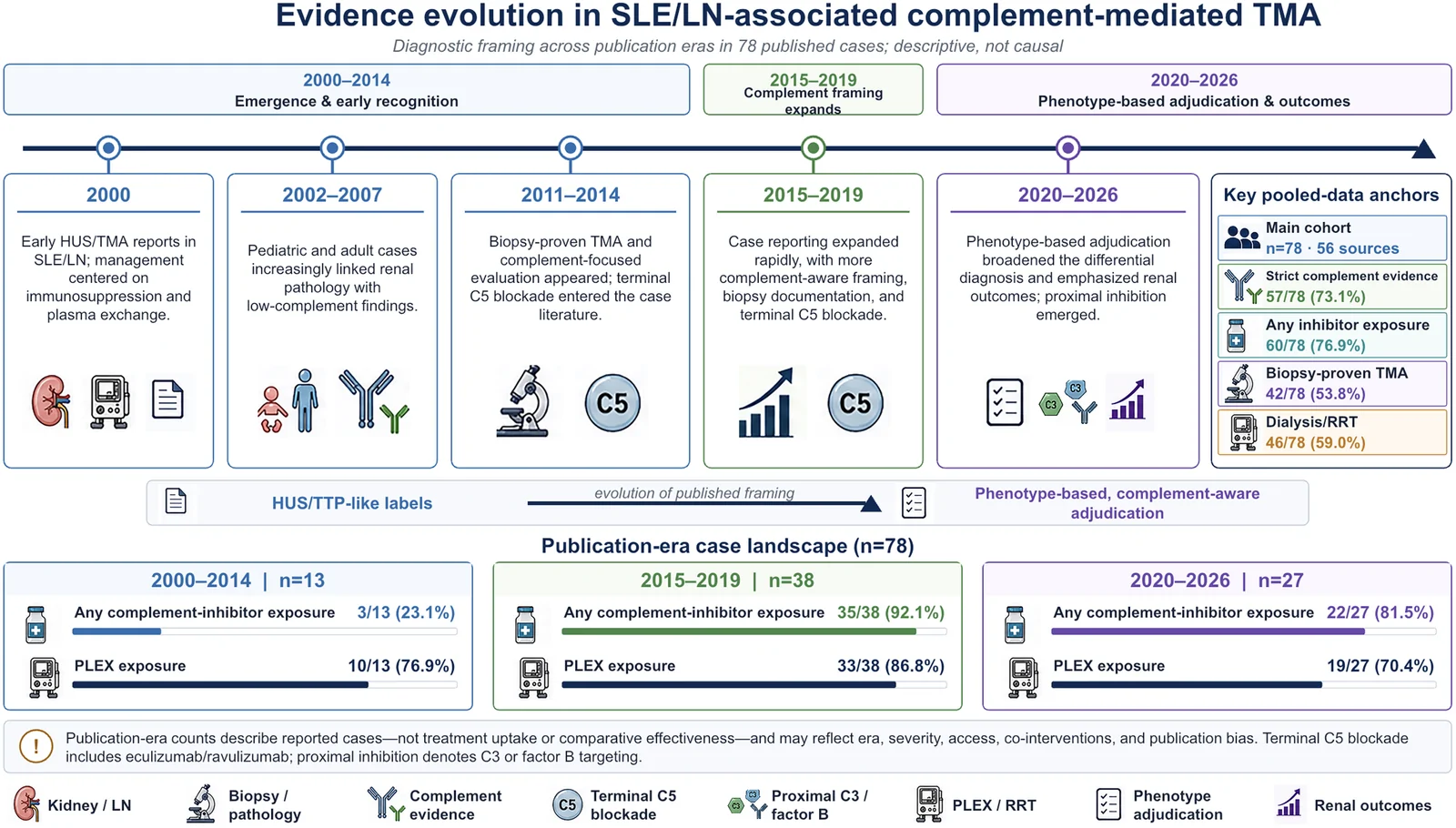
Evidence evolution and diagnostic framing in systemic lupus erythematosus (SLE)/lupus nephritis (LN)-associated complement-mediated thrombotic microangiopathy (TMA). The pooled data anchors and publication era summaries derive from the frozen 78-patient, 56-source dataset: strict complement-related evidence, 57/78; any complement inhibitor exposure, 60/78; biopsy-proven TMA, 42/78; and dialysis/renal replacement therapy (RRT), 46/78. The era groups comprised 13 patients in 2000–2014, 38 in 2015–2019, and 27 in 2020–2026. Complement inhibitor exposure was reported in 3/13, 35/38, and 22/27, and plasma exchange (PLEX) was reported in 10/13, 33/38, and 19/27, respectively. Terminal C5 blockade includes eculizumab or ravulizumab. Proximal inhibition refers to C3- or factor B-targeted therapy. Era-specific frequencies are descriptive and should not be interpreted as treatment uptake or comparative effectiveness.

**Figure 6:**
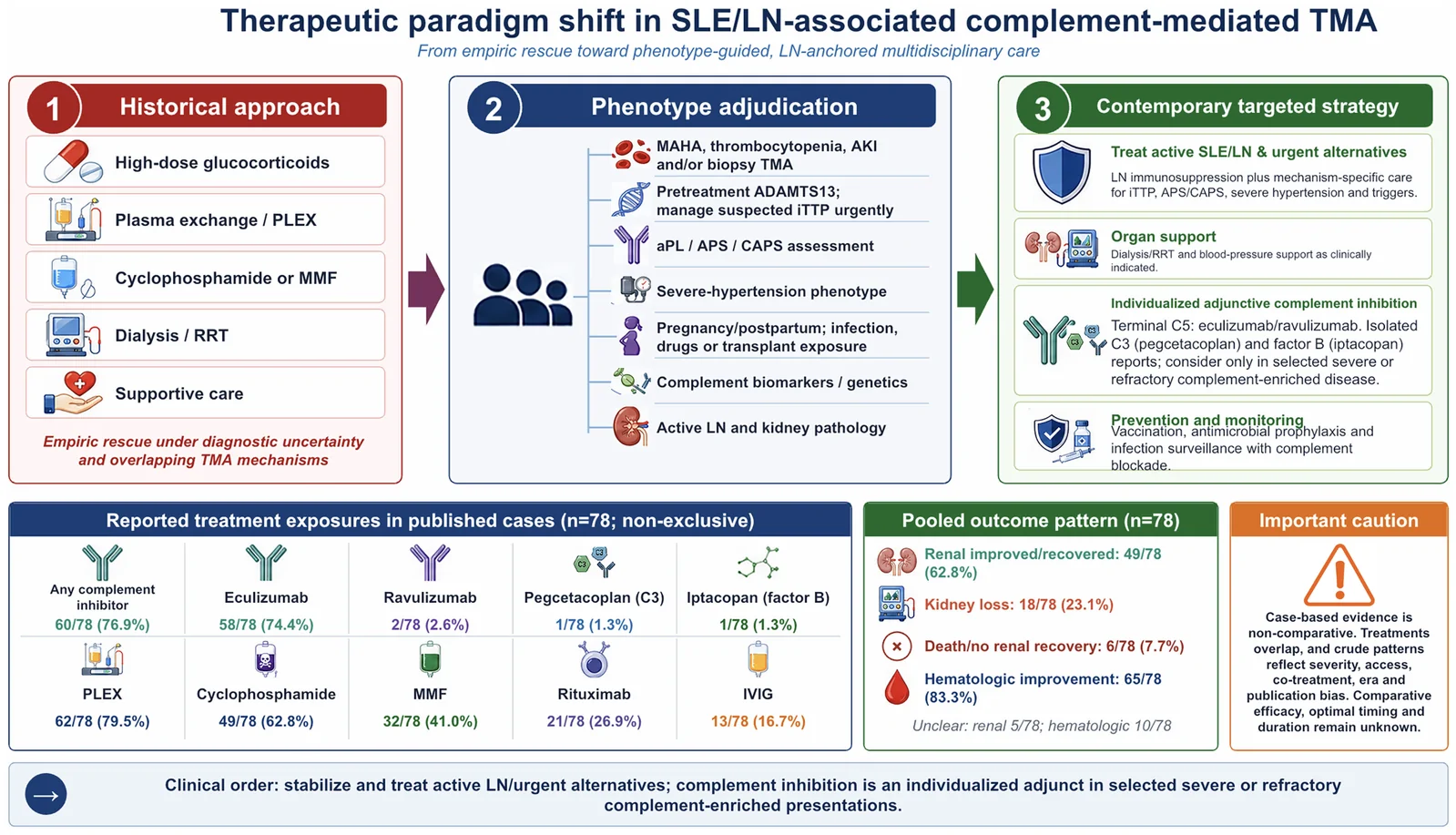
Therapeutic paradigm shift in systemic lupus erythematosus (SLE)/lupus nephritis (LN)-associated complement-mediated TMA. Historical rescue approaches are shown for context. Phenotype adjudication emphasizes confirmation of thrombotic microangiopathy (TMA), urgent pretreatment ADAMTS13 assessment, competing mechanism evaluation, active LN and kidney pathology, and complement-related evidence. In the contemporary strategy, evidence-based LN treatment, alternative-specific management, and organ support precede the consideration of complement inhibition. Terminal C5 blockade includes eculizumab or ravulizumab. C3 inhibition and factor B inhibition were represented by isolated case reports. Complement inhibition is therefore an individualized adjunct for selected severe or refractory complement-enriched presentations, not an established first-line therapy. *Lower panels* summarize the non-exclusive treatments and descriptive outcomes from 78 patients, and these uncontrolled data do not establish comparative efficacy, optimal timing, duration, or a universal sequence.

The complement-related evidence domains reported in *Section 3.4* are summarized separately in **Figure 7**. Complement inhibitor exposure was included only as a therapeutic context and not as a mechanistic proof.

**Figure 7:**
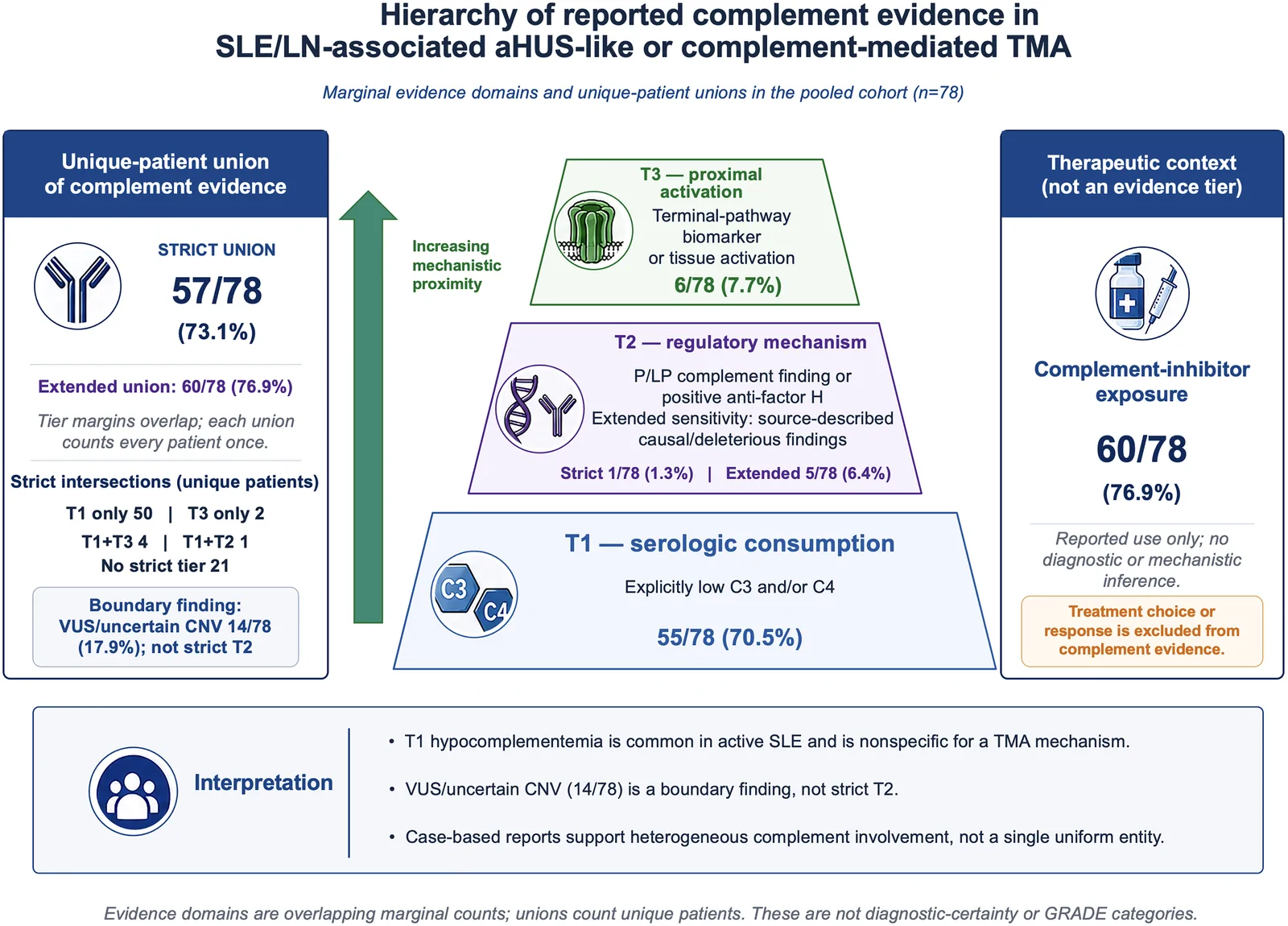
Graded reported complement-related evidence in systemic lupus erythematosus (SLE)/lupus nephritis (LN)-associated thrombotic microangiopathy (TMA). *T1* denotes explicitly low C3 and/or C4 levels (55/78); *strict T2* denotes a pathogenic/likely pathogenic complement finding or a positive anti-factor H antibody result (1/78), with an extended sensitivity definition in 5/78; and *T3* denotes a terminal pathway biomarker or a tissue complement-activation signal documented during the index episode (6/78). The strict, non-mutually exclusive union was 57/78, and the extended union was 60/78. Variants of uncertain significance (VUS)/uncertain findings (14/78) were not counted as strict T2. Complement inhibitor exposure (60/78) is a therapeutic context, not a mechanistic proof.

### 3.6 Reported renal and hematologic outcomes

Renal improvement/recovery or dialysis independence were reported in 49 patients (62.8%), persistent kidney function loss/dialysis dependence in 18 (23.1%), death without renal recovery or explicit no recovery in 6 (7.7%), and an unclear renal outcome in 5 patients (6.4%). Among 73 patients with a classifiable renal outcome, 49 (67.1%) improved. Hematologic improvement was reported in 65 patients (83.3%), no response in 3 (3.8%), and an unclear outcome in 10 (12.8%). Among the 68 evaluable patients, 65 (95.6%) improved. These *post-hoc* categories combine heterogeneous endpoints and follow-up intervals.

In the no-inhibitor class, the renal outcomes in the 18 patients were improvement in 15, kidney loss in one, death/no recovery in one, and unclear in one. In the eculizumab-only class, they were 32, 15, 5, and 4 out of 56 patients, respectively. In the four other complement sequences, two improved, and two had kidney function loss. The hematologic outcomes were improvement/no response/unclear in 15/2/1, 46/1/9, and 4/0/0 patients, respectively (**Figure 3**). These differences are not treatment effects; therapies overlapped, and the severity, timing, access, chronic kidney damage, and follow-up differed.

### 3.7 Full-text, definition, authorship, and patient-level sensitivity analyses

The full-text-only subset contained 61 patients. Compared with the entire cohort, the full-text subset had strict complement-tier evidence in 47/61 (77.0% vs. 57/78, 73.1%), complement inhibitor exposure in 43/61 (70.5% vs. 60/78, 76.9%), renal improvement in 41/61 (67.2% vs. 49/78, 62.8%), and hematologic improvement in 54/61 (88.5% vs. 65/78, 83.3%). Excluding published abstracts did not reverse the descriptive signals, but could not remove publication bias or heterogeneity among full texts.

Replacing strict with extended T2 changed the complement union from 57 to 60 patients. Excluding the author-affiliated Yu case yielded 77 patients from 55 sources: T1 in 54, T3 in 5, strict complement union in 56, any complement inhibitor in 59, renal improvement in 48, and hematologic improvement in 64. No qualitative conclusions changed. This analysis addresses non-systematic inclusion risk, not the broader publication bias.

We generated **Figure 8** as a deterministic 78-row visualization from the same patient-keyed analytical table used for **Supplementary Table S5**. We used the source table’s stable patient keys and source links for programmatic checks and labeled the displayed rows Case 1–Case 78 after sorting by publication era and stable patient key. We checked the four-state phenotype fields (present, absent, not reported, and unclear), treatment exposures, outcomes, and marginal totals programmatically. The index class strip uses a separate palette from treatment exposure, while the labeled era strip provides ordering context. No image-generating model was used.

**Figure 8:**
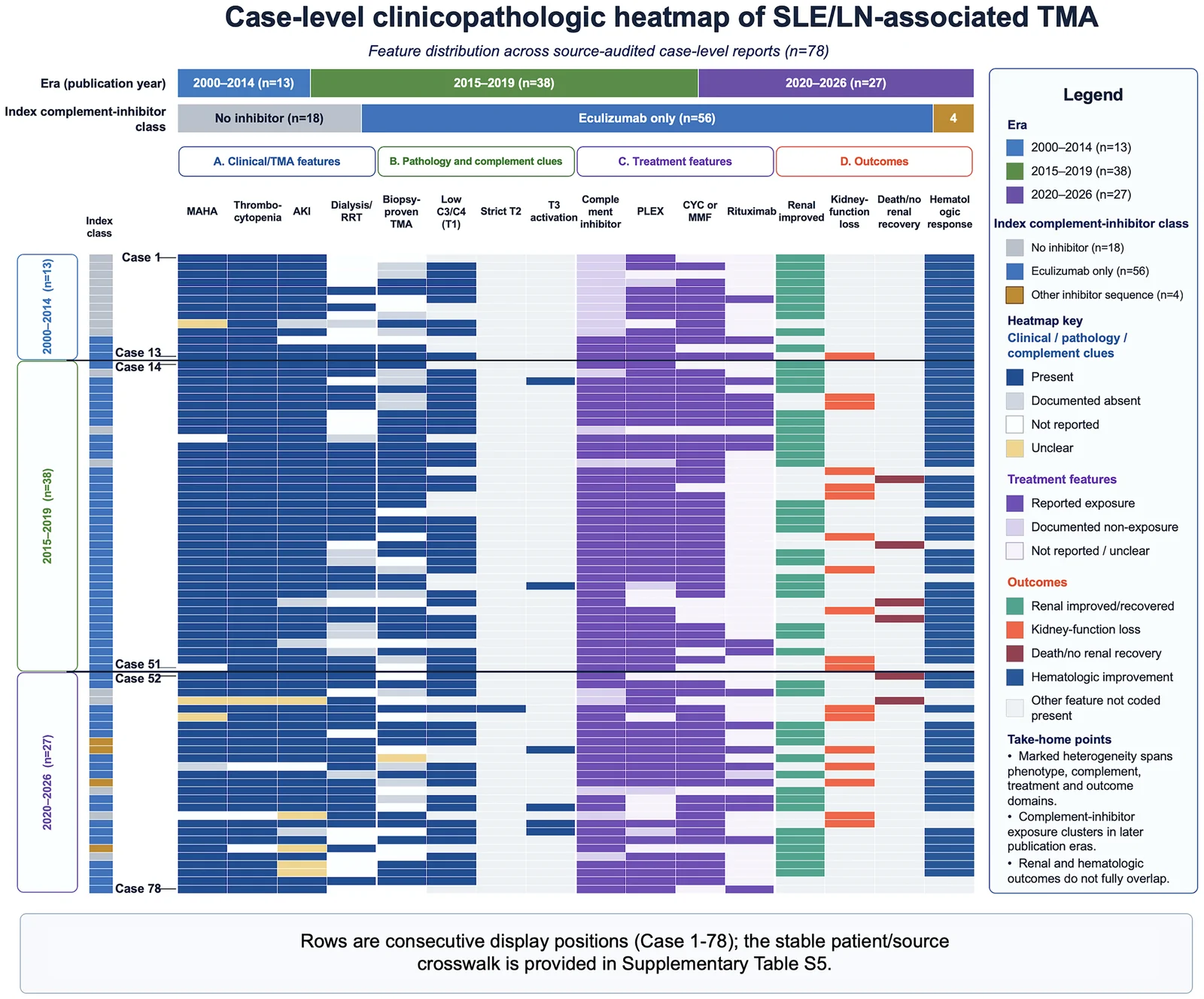
Case-level clinicopathologic heatmap of systemic lupus erythematosus (SLE)/lupus nephritis (LN)-associated thrombotic microangiopathy (TMA). The displayed rows are consecutively labeled *Case 1*–*Case 78* after ordering by publication era and stable patient key; **Supplementary Table S5** retains the stable patient/source crosswalk. Five source-adjudicated phenotype columns distinguish present, documented absent, not reported, and unclear. *Light cells* in other domains mean “not coded present” and must not be interpreted as proven absence. Separate annotations show era, mutually exclusive index complement inhibitor class, complement-related evidence, reported treatments, and outcomes. The matrix is descriptive, not a clustering or causal analysis.

### 3.8 Reporting completeness and GRADE-informed certainty

The 39 full-text sources comprised 32 single-case reports/letters and seven case series. A project-specific completeness summary classified 35 reports as higher-completeness and four as moderate-completeness. These are descriptive categories rather than official JBI grades. The 17 published abstract sources (16 conference abstracts and one peer-reviewed journal case available only at the abstract level) were evaluated separately and had substantial limitations in diagnostic detail, treatment timing, follow-up, outcome definition, and harms (**Table 4**).

**Table 4:** Source-level reporting completeness and critical appraisal summary.

Domain	Metric	Reported	Interpretation
Source availability	Full text available	39/56	Published abstracts retained as a lower-information stratum
Patient-level availability	Full-text patient rows	61/78	Abstract-only rows were not treated as negative
Differential diagnosis	ADAMTS13/TTP assessment reported	55/78	Incomplete exclusion of immune TTP in remaining reports
Kidney pathology	Kidney pathology reported	71/78	Pathology terminology and sampling timing varied
Outcomes	Renal outcome classifiable	73/78	Five retained as unclear
Outcomes	Hematologic outcome evaluable	68/78	Ten retained as unclear
Selection bias	Case series consecutive inclusion	0/7	Selection methods usually unclear
Safety	Adverse event reporting	Not estimable	Too incomplete for an incidence estimate

The JBI domain assessment and project completeness categories characterize reporting, not evidence certainty. One reviewer conducted the JBI appraisal, and we did not perform a duplicate human appraisal of every domain.

TTP, thrombotic thrombocytopenic purpura.

The GRADE-informed certainty was very low for reported renal improvement, hematologic improvement, kidney function loss, mortality/no recovery, safety/harms, and complement–mechanistic associations. Serious risk of bias and publication bias were inherent to the selectively published cases. Inconsistency, indirectness, and imprecision were also serious. The comparative effects of complement inhibition *versus* no inhibition, individual agents, PLEX, CNI, antithrombotic therapy, or immunosuppressive regimens were not estimable (**Table 5**).

**Table 5:** GRADE-informed certainty profile for key findings.

Outcome/question	Evidence base	Finding	Certainty	Main reasons for certainty judgment	Interpretation
Renal improvement after reported management	78 case-based patients	49/78	Very low	Uncontrolled, selected reports; heterogeneous co-interventions/endpoints	Descriptive frequency only; no causal inference
Kidney loss/dialysis dependence	78 case-based patients	18/78	Very low	Variable follow-up; death and chronic damage compete with recovery	Descriptive burden among published cases
Hematologic improvement	78 case-based patients	65/78	Very low	Outcome definitions/timing inconsistent; 10 unclear	Laboratory response cannot substitute for renal outcome
Comparative effect of complement inhibition	No randomized or mechanism-matched comparison	Not estimable	Very low	Critical confounding by indication, era, access, co-treatment, publication bias	Do not calculate a pooled relative effect
Complement biomarker/genetic enrichment	Non-mutually exclusive patient-level evidence	Strict union 57/78	Very low	T1 nonspecific; testing selective and assays evolved	Supports heterogeneity, not diagnosis
Treatment harms	Case reports/abstracts	Not estimable	Very low	Selective and sparse safety reporting	Use established class guidance and prospective surveillance

Certainty applies to the descriptive findings among the published cases and cannot be interpreted as population incidence or comparative treatment effect. “Not estimable” indicates absence of a credible counterfactual comparison, not evidence of no effect.

### 3.9 Representative kidney pathology and evidentiary boundary

**Figure 9** uses the author-affiliated Yu 2026 case to illustrate the clinicopathologic complexity of proliferative/membranous LN occurring with a systemic TMA phenotype (89). The light and electron microscopy images principally demonstrate immune complex LN. They do not show a pathognomonic complement lesion and are not used as independent proof of renal TMA or complement-mediated TMA. The diagnosis in that report depended on the integrated clinical phenotype, non-severely deficient ADAMTS13, soluble C5b-9 elevation, pathology, and course. We did not use figure selection as an eligibility criterion, and the exact selection date was unavailable.

**Figure 9:**
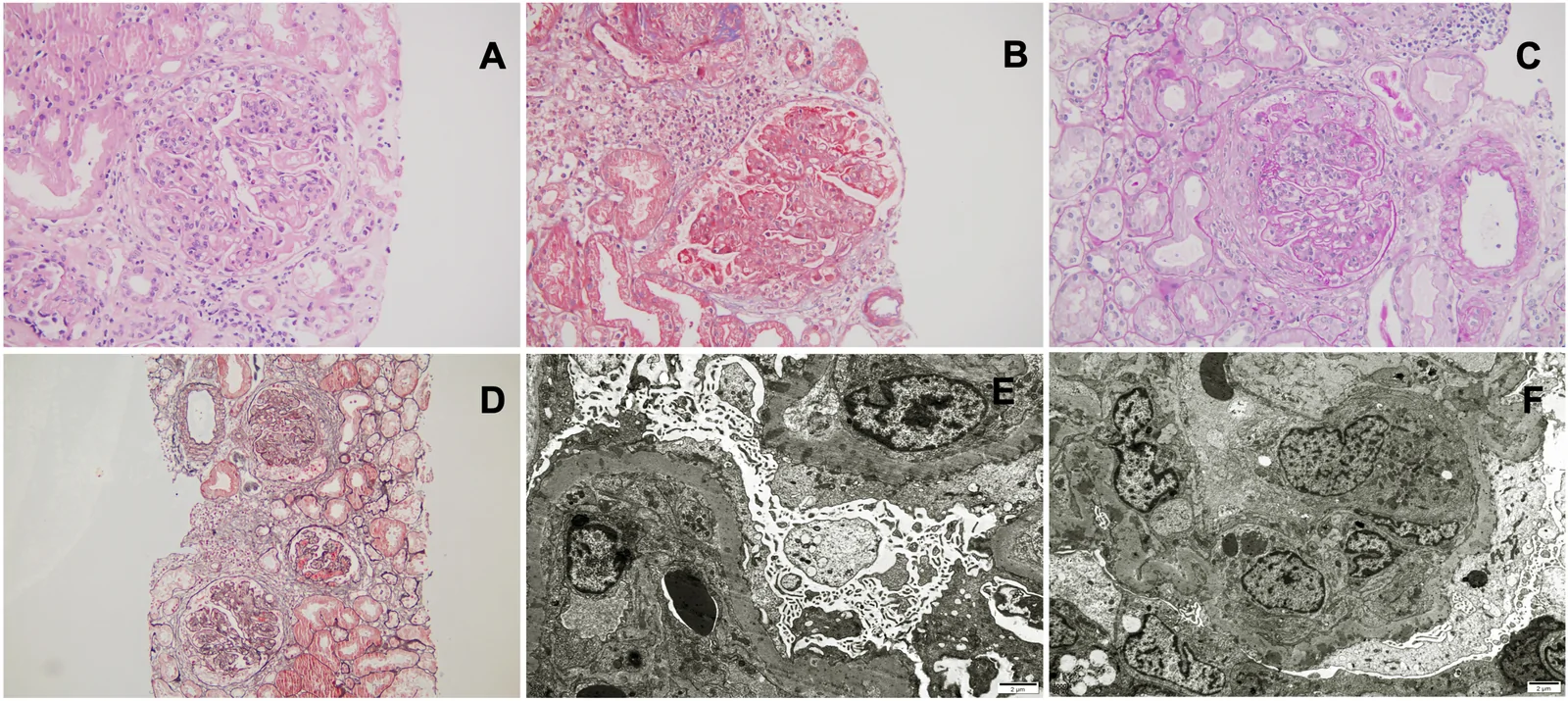
Representative kidney biopsy findings from an author-affiliated included case of class IV+V lupus nephritis. **(A)** H&E, ×200: mesangial and endocapillary hypercellularity. **(B)** Masson trichrome stain, ×200: fuchsinophilic deposits with segmental capillary wall thickening. **(C)** PAS, ×200: proliferative glomerular lesion. **(D)** PASM–Masson, ×100: tubular epithelial injury with focal interstitial inflammation. **(E)** TEM, ×5,000, 60 kV: glomerular basement membrane wrinkling/thickening as reported in the source. **(F)** TEM, ×4000, 60 kV: mesangial cellular and matrix expansion. *Scale bars* in **(E, F)**, 2 μm. These panels principally illustrate immune complex lupus nephritis. No panel shows an unequivocal diagnostic thrombus or independently establishes renal thrombotic microangiopathy (TMA) or a complement-mediated mechanism. Adapted from Yu et al. under the article’s Creative Commons Attribution 4.0 license (89).

## 4 Discussion

### 4.1 Principal findings and inference boundary

This systematic review defines SLE/LN-associated TMA as a recognizable but heterogeneous high-risk phenotype rather than a single disease. Across 56 sources and 78 patients, the dominant pattern combined thrombocytopenia, kidney injury, hemolysis, and renal microvascular injury; however, no feature was universal. Strict biopsy TMA was present in 42 patients, and RRT was required in 46. Microvascular injury should be considered when kidney dysfunction is disproportionate to conventional LN activity, while recognizing that the case literature cannot determine its frequency in an unselected SLE/LN population.

The central mechanistic result is not simply that complement abnormalities were frequent, but that specificity collapsed under rigorous definitions. Explicit low C3/C4 levels were common, whereas only one patient met strict T2 and six had direct terminal/tissue activation at the index event. Four additional source-attributed genetic findings entered extended T2 only, and 14 remained uncertain. The exact overlap analysis prevents the tiers from being mistaken for mutually exclusive or additive groups.

Treatment findings require still greater restraint. Complement inhibitors were reported in 60 patients, nearly always within multimodal regimens. Renal and hematologic improvements were frequently described, but with class differences being dominated by indication, timing, co-interventions, access, era, and selective publication. There is no population denominator, valid untreated counterfactual, or adequate safety ascertainment. We therefore make no claim about incidence, prognosis, response probability, superiority, or optimal duration.

### 4.2 Complement as driver, amplifier, or epiphenomenon

The conventional distinction between “primary complement-mediated aHUS” and “secondary TMA” is too rigid for many patients with SLE. Contemporary complement biology supports a layered model in which inherited or acquired alternative pathway dysregulation interacts with disease-specific triggers, endothelial context, and tissue injury (15, 16). Complement can be a primary driver in a patient with a pathogenic regulatory defect or functionally important autoantibody, an amplifier that converts lupus-associated endothelial stress into self-sustaining injury, or an epiphenomenon generated by severe inflammation and tissue damage without being the dominant therapeutic target (20–22).

SLE provides several routes into this loop. Immune complexes activate complement and consume circulating components, while nucleic acid sensing and type I interferon programs prime vascular inflammation. Abnormal neutrophil subsets injure the endothelium, impaired NET clearance prolongs exposure to nuclear material, and oxidized mitochondrial DNA reinforces interferogenic inflammation (20–22). Platelet CD154 can augment plasmacytoid dendritic cell interferon production (23). At the complement–coagulation interface, C5a receptor signaling can enhance neutrophil tissue factor activity, providing a route by which complement may intensify thrombosis rather than merely accompany it (24). These mechanistic studies support plausibility, but do not establish the initiating mechanism in a case report.

LN adds a tissue-specific second hit. Immune complex deposition, glomerular inflammation, arteriolar injury, hypertension, ischemia, and local complement activation occur within a vascular bed exposed to circulating inflammatory and thrombotic mediators. APS, pregnancy, infection, medications, or severe blood pressure elevation add further stress. A phenotype can therefore become complement-dependent without a monogenic disorder. Conversely, complement deposition or soluble activation products may simply mark the injury severity. Low C3/C4 levels cannot discriminate these states.

Negative genetic testing does not exclude complement amplification because panels are incomplete, penetrance is variable, acquired mechanisms occur, and activation can be transient or tissue-restricted (15, 16). Conversely, a common polymorphism, VUS, or copy number change cannot establish causality without population frequency, segregation, functional, and contemporary classification evidence (17, 18). Treatment response is likewise non-diagnostic: interruption of a downstream amplifier may improve a secondary process without proving that complement initiated it. “Complement-amplified TMA” is therefore a testable hypothesis, not a synonym for inherited aHUS.

### 4.3 Bridging clinical TMA and kidney pathology

Renal pathology is indispensable, but answers a morphologic rather than an etiologic question. LN vascular lesions include immune deposits, arteriosclerosis, non-inflammatory necrotizing vasculopathy, vasculitis, APS-associated lesions, and acute or chronic TMA. Incorporating vascular lesions improves the prognostic characterization beyond glomerular class (5). This is particularly relevant when severe dysfunction, hypertension, or dialysis dependence is not adequately explained by the glomerular activity index.

Acute TMA lesions can include endothelial swelling, luminal narrowing, fibrin or platelet thrombi, mesangiolysis, and fragmented erythrocytes. Chronic injury may manifest as basement membrane duplication, fibrous intimal thickening, recanalization, or persistent ischemic remodeling. Active endothelial injury may remain reversible, and cortical necrosis, established chronic TMA, and extensive interstitial fibrosis constrain kidney recovery even when hemolysis and thrombocytopenia resolve. Biopsy timing can therefore influence the diagnosis and apparent response.

LN biopsy cohorts generally identify renal TMA as a marker of more severe presentation; however, independent prognostic estimates vary with definitions, treatment, baseline function, activity, and chronicity (6, 8–10). Complement staining supports biological plausibility in some cohorts, but is not sufficiently specific to identify a treatment-responsive subgroup (8). The absence of a visible thrombus does not exclude TMA because lesions are focal and evolve. Clinical and biopsy phenotypes should therefore be linked without being collapsed: biopsy establishes microvascular injury, while etiology requires context, ADAMTS13, APS testing, complement evaluation, exposures, and chronology.

### 4.4 A phenotype-first differential diagnosis

A phenotype-first approach confirms the syndrome and identifies urgent alternatives before assigning an aHUS label. The initial questions are whether hemolysis is microangiopathic, thrombocytopenia reflects consumption rather than immune destruction or medication, organ injury is acute, and the process is systemic or kidney-predominant. Blood smear, lactate dehydrogenase, haptoglobin, bilirubin, reticulocytes, platelet trajectory, coagulation tests, creatinine, urinalysis, blood pressure, and organ assessment should be interpreted together.

Immune TTP is the most urgent competing diagnosis. ADAMTS13 should be sampled before plasma therapy whenever possible without delaying treatment, and empirical TTP-directed treatment remains appropriate when probability is high (90). ADAMTS13/TTP assessment was reported in only 55 of 78. Missing or post-treatment assays limit certainty, and suggestive wording alone cannot retrospectively establish severe deficiency.

APS/CAPS require parallel consideration because SLE, thrombosis, thrombocytopenia, pregnancy morbidity, and renal microangiopathy frequently overlap. The classification criteria standardize profiles and events, but do not substitute for causal adjudication (91). Persistent lupus anticoagulant, anti-cardiolipin or anti-β2-glycoprotein I antibodies, macrovascular thrombosis, obstetric history, and multi-organ thrombosis shift probability. The APS/CAPS wording in 25 patients indicates overlap, not exclusive causation.

Pregnancy compresses the differential among preeclampsia/HELLP, TTP, and complement-mediated TMA. The gestational timing, liver injury, placental disease, persistence after delivery, ADAMTS13, and kidney trajectory are more informative than a label (11). Severe hypertension can itself produce hemolysis and renal failure. Improvement with pressure control supports this mechanism, but does not exclude complement susceptibility (12). Infection and DIC require coagulation, microbiology, and organ-context assessment (13). Drug exposure should be reconstructed because immune- and dose-dependent drug-induced TMA differ (14). Active LN remains both an explanation and a trigger, and LN-directed therapy is essential when immune complex activity is present (1, 2, 4). The practical output should be a structured probability statement with coexisting labels rather than a forced single diagnosis.

### 4.5 Interpreting complement assays and genetics

Complement testing is most useful as a hierarchy tied to sampling time. C3/C4 tests are widely available, but nonspecific, and normal concentrations do not exclude local or transient alternative pathway activation. CH50/AH50 assess pathway integrity rather than identify the cause. Soluble terminal products and split products can indicate activation, but the assays, transport, reference ranges, and timing vary. PLEX removes components and autoantibodies, and C5 inhibitors alter terminal pathway functional assays. Tissue staining localizes deposition, but cannot distinguish the cause, amplification, and consequence. Pretreatment and serial measurements are preferable (16, 92).

Each genetic variant should be reported with transcript, nucleotide/protein change, zygosity, laboratory classification, population frequency, functional evidence, and interpretation date. The ACMG/AMP standards separate pathogenic, likely pathogenic, uncertain, likely benign, and benign variants (18). VUS should not diagnose complement-mediated TMA or justify indefinite therapy. Anti-factor H antibodies are acquired and require assay provenance, titer, and timing (19). Copy number and hybrid gene testing may require methods beyond routine sequencing; however, an uncharacterized “mutation” remains uncertain, not presumptively pathogenic.

**Figure 7** therefore shows graded, overlapping evidence domains rather than a “highest tier” diagnosis. Conventional serum consumption provides low-specificity context, strict T2 supports a regulatory mechanism when properly classified, and T3 demonstrates pathway engagement without proving initiation. Treatment choice and response are displayed only as context because they are not mechanistic tests.

### 4.6 A mechanism-matched therapeutic framework

Treatment proceeds in parallel layers (**Figure 6**). The first is stabilization and prevention of irreversible injury: obtain pretreatment samples when feasible, support organs, control blood pressure, treat infection, initiate dialysis when needed, and provide empirical TTP therapy when probability is high. The second addresses demonstrated drivers: active LN requires guideline-concordant immunosuppression; APS/CAPS require appropriate antithrombotic and immunomodulatory management; offending drugs are withdrawn; and pregnancy-associated syndromes require coordinated obstetric, hematologic, nephrologic, and critical-care decisions (4, 11, 90, 91). Complement blockade does not displace these actions.

PLEX has different meanings across mechanisms. It is lifesaving in immune TTP and can remove pathogenic antibodies in selected immune or complement autoantibody states; however, its use in undifferentiated TMA does not establish TTP, and non-response does not prove complement-mediated disease. PLEX was reported in 62 patients, generally alongside corticosteroids, cyclophosphamide, rituximab, dialysis, or complement inhibitors. Sequence, indication, and timing—not isolated exposure—are the appropriate units of interpretation.

Complement inhibition becomes most defensible when TMA is severe or progressive, urgent alternatives are addressed, LN treatment is underway, and evidence suggests complement enrichment. Relevant features can include persistent microangiopathy despite control of lupus activity, active biopsy TMA, a pathogenic complement defect, anti-factor H autoimmunity, reproducible terminal activation, compatible relapse, or a trigger that exposes susceptibility. None is individually sufficient. The decision integrates severity, reversibility, competing mechanisms, test quality, infection risk, access, and treatment burden.

Eculizumab produced hematologic and renal responses in prospective trial-defined aHUS cohorts, establishing terminal C5 blockade as a therapeutic principle (93). Ravulizumab extends the dosing interval and has been studied in adult and pediatric aHUS cohorts (94, 95). These studies are external precedent, not evidence that C5 inhibition is universally effective in SLE/LN-associated TMA.

Proximal inhibitors are mechanistically distinct. Iptacopan inhibits factor B, and its evidence in other complement diseases supports pathway feasibility, but not efficacy in lupus TMA (96). Pegcetacoplan inhibits C3. The phase 2 DISCOVERY basket study (NCT03453619) evaluated pegcetacoplan in complement-mediated kidney diseases and included an LN stratum, but was not a trial of SLE/LN-associated TMA and cannot be used to infer benefit here (97). The one pegcetacoplan- and the one iptacopan-exposed report in this review are hypothesis-generating only. No obinutuzumab-treated eligible TMA case was found. Obinutuzumab is B-cell-directed, not a complement inhibitor, and its absence from these selected reports is not evidence about LN practice.

Timing may be as important as agent. Complement blockade cannot reverse cortical necrosis, advanced chronic TMA, or extensive fibrosis, but waiting for complete genetics can sacrifice recoverable kidney function in a rapidly progressive complement-enriched phenotype. Prospective studies should record intervals from TMA recognition to PLEX, immunosuppression, complement inhibition, dialysis, hematologic response, and kidney recovery.

Safety is integral. Terminal complement inhibition markedly increases susceptibility to invasive meningococcal disease, and breakthrough infection occurs despite vaccination (98). Patients should receive age-appropriate MenACWY and MenB vaccination and boosters under current local guidance (99). When urgent treatment cannot await vaccine response, antimicrobial prophylaxis and rapid-access infection plans are required according to applicable recommendations. Prophylaxis does not eliminate risk and may select less susceptible organisms (100). Every report should document vaccination, prophylaxis, education, infection, and other harms.

Duration remains unresolved even in primary aHUS. Relapse after eculizumab withdrawal is associated with pathogenic variants in some cohorts (101). Structured withdrawal and rapid retreatment can be feasible in selected primary aHUS populations, but high-risk exclusions and different triggers limit transferability to lupus (102). In SLE/LN, decisions additionally depend on the trigger persistence, kidney recovery, residual TMA, pathogenic variants/autoantibodies, relapse history, pregnancy plans, adherence, access, and monitoring capacity.

### 4.7 Renal–hematologic discordance is clinically meaningful

Hematologic improvement was reported in 65 of 78 and renal improvement in 49 of 78. This should not be reduced to a generic “response rate.” Platelets and hemolysis can improve rapidly once consumption abates, whereas kidney recovery depends on the ischemia duration, dialysis exposure, LN activity, vascular chronicity, cortical necrosis, fibrosis, and treatment delay. Hematologic remission can coexist with dialysis dependence.

“Complete response” should therefore contain separate hematologic and renal domains. Reports should provide time to platelet normalization, lactate dehydrogenase and haptoglobin trajectories, creatinine/eGFR, proteinuria, dialysis dates, and kidney status at standardized intervals. Death without renal recovery must remain distinct from persistent kidney failure among survivors. Patient-important outcomes include dialysis independence, relapse, treatment toxicity, infection, pregnancy outcome, and quality of life.

### 4.8 Relationship to denominator-defined LN and SLE cohorts

Case reports and LN cohorts answer complementary questions. Cases are enriched for rare findings, severe disease, rescue therapy, and apparent success. They permit sequence reconstruction, but cannot estimate incidence or effect. Biopsy cohorts begin with a broader LN denominator and assess prevalence and prognosis, but often lack detailed ADAMTS13, genetics, terminal biomarkers, or rescue timing.

Across established cohorts, renal TMA generally marks more severe presentation and impaired kidney recovery, although independence varies after adjustment (5–10). Three aggregate reports identified during the update—124 patients with SLE-TMA, 78 TMA lesions among 644 LN biopsies, and 22 children with LN-TMA—could not enter the patient-level synthesis, but strengthen the denominator-defined contextual evidence (103–105). Their aggregate comparisons should be analyzed on their own designs, not mixed with case reports or used to retroactively infer efficacy in this dataset.

The next advance requires linkage of both streams: standardized pathology, pretreatment complement biomarkers, differential testing, exact treatment timing, and longitudinal outcomes within a defined SLE/LN denominator. Candidate phenotypes include immune complex LN with secondary microvascular injury, APS-associated TMA, systemic TMA superimposed on LN, and a complement-enriched subgroup with sustained pathway activation. These should be tested prospectively rather than imposed from treatment choice.

### 4.9 Pediatric and pregnancy-associated disease

There were 11 patients who were younger than 18 years and four with pregnancy/postpartum context. These small numbers cannot support subgroup estimates. Pediatric disease raises questions about genetic susceptibility, vaccination, cumulative infection risk, growth, treatment burden, and kidney reserve. The ravulizumab evidence in pediatric primary aHUS and the aggregate LN-TMA cohort are external context, not proof for pediatric lupus TMA (95, 105).

Pregnancy changes complement, coagulation, blood pressure, and endothelial physiology, while SLE/APS adds risk. The gestational age, placental findings, liver involvement, ADAMTS13, anti-phospholipid antibodies, postpartum persistence, and response to delivery are essential (11). Treatment decisions must consider maternal urgency, fetal exposure, vaccination, infection risk, breastfeeding, and recurrence. These variables were inconsistently reported and should be mandatory in future registries.

### 4.10 Minimum dataset and future causal designs

A minimum dataset should contain five linked modules. The *phenotype module* records the dates of SLE, LN, and TMA onset; blood count/hemolysis trajectories; organs; blood pressure; pregnancy; infection; and drug triggers. The *differential module* contains the pretreatment ADAMTS13/inhibitor, anti-phospholipid profile, coagulation, microbiology, Shiga toxin testing where relevant, and transplant/malignancy context. The *kidney module* captures serial creatinine, proteinuria, dialysis dates, biopsy timing, LN class/activity/chronicity, and standardized acute/chronic TMA lesions. The *complement module* reports C3/C4, CH50/AH50, terminal markers, tissue staining, anti-factor H, panel content, variant-level ACMG/AMP classification, and sampling relative to PLEX/inhibition. The *treatment outcome module* records the exact sequence, dose, timing, prophylaxis, adverse events, domain-specific responses, withdrawal, relapse, and common follow-up intervals.

A prospective multicenter registry should enroll all SLE/LN patients evaluated for TMA, not only those receiving complement blockade. Nested phenotypes should be adjudicated by a blinded multidisciplinary panel. Pretreatment biospecimens would permit centralized functional assays, and pathology should be re-reviewed with standardized vascular lesion definitions blinded to treatment/outcome.

Future causal analyses require more than larger numbers. Severity, treatment failure, diagnostic confidence, era, and access influence both complement blockade and outcome—classic confounding by indication (106). A target trial framework should define eligibility, time zero, strategies, assignment, follow-up, outcomes, contrast, and analysis before comparing early complement blockade with mechanism-matched alternatives (107). Alignment of eligibility and initiation avoids immortal-time and survivor bias. Until such data exist, transparent synthesis without meta-analysis is preferable to manufactured precision (32).

### 4.11 Strengths and limitations

Strengths include patient-level reconstruction, explicit source/patient denominators, canonical source linkage, verification of attachment identity and source eligibility, distinction between full texts and published abstracts, four-state phenotype coding, non-mutually exclusive complement tiers with exact overlap, separation of VUS from strict T2, administration of treatment coding with “not reported” retained, patient-keyed renal and hematologic outcomes, sensitivity analyses, a GRADE-informed profile, and deterministic generation of data-dependent figures from the frozen 78-row dataset.

The limitations are decisive.

1. Publication and selection bias. Case reports are selected for novelty, diagnostic complexity, rescue therapy, and apparent success, and failed, untreated, mild, or routine experiences are likely underrepresented. No incidence, prognosis, response probability, or safety rate can be estimated.
2. Protocol and timing deviations. The identification search preceded PROSPERO registration, the registered CNKI search could not be verified and was not included, and the August update was PubMed-only rather than a common date rerun of all databases. Language and database bias therefore remain possible.
3. Single-reviewer conduct. Only one reviewer conducted screening, extraction, and JBI appraisal, contrary to the registered plan. Computationally assisted source and data verification did not constitute dual-independent human screening or dual-human extraction.
4. Abstract-level evidence. Of the published abstracts, 17 form a lower-information layer and are not evidentially equivalent to full-text reports.
5. Changing definitions and tests. The SLE/LN criteria, aHUS terminology, ADAMTS13 availability, gene panels, and variant standards changed from 2000 to 2026.
6. Missingness and *post-hoc* harmonization. ADAMTS13/TTP assessment was reported in 55 of 78 and pathology in 71 of 78. The follow-up, treatment timing, and safety were inconsistent.
7. No causal treatment comparison. Co-interventions, confounding by indication, access, calendar time, survivor selection, and outcome reporting bias invalidate crude contrasts.
8. Author-affiliated source. Eight review authors co-authored Yu 2026. We report its other-methods identification, authorship relationship, and leave-one source-out analysis.

These constraints mean that the review can map a phenotype, organize mechanistic evidence, and establish a research framework, but cannot validate the diagnostic criteria, quantify the population risk, prove the treatment benefit, or define a universal duration.

## 5 Conclusion

SLE/LN-associated TMA is a heterogeneous, kidney-threatening phenotype in which immune complex activity, APS, pregnancy, infection, hypertension, drugs, and complement dysregulation may coexist. The 78 reported patients are consistent with a driver–amplifier–epiphenomenon model rather than automatic classification as either nonspecific secondary TMA or primary aHUS. Low C3/C4 levels, biopsy TMA, and treatment response are insufficient mechanistic proof, and ADAMTS13, competing causes, functional complement studies, variant-level interpretation, pathology, and chronology must be integrated.

Complement blockade has biological precedent in trial-defined aHUS and may be considered for selected complement-enriched SLE/LN presentations that are severe, progressive, or refractory, after or alongside urgent alternative-specific and LN-directed care. The current case-based evidence—rated very low certainty and profoundly vulnerable to publication bias—does not establish the incidence, prognosis, comparative efficacy, optimal timing, agent selection, safety rate, or duration. Progress depends on prospective denominator-defined multicenter cohorts, pretreatment biospecimens, standardized pathology, explicit treatment sequences, domain-specific outcomes, safety surveillance, and causal designs that address confounding by indication.

## Data Availability

The deidentified patient extraction matrix, source crosswalk, data dictionary, protocol-deviation log, analysis summary, deterministic figure code, and QC manifest supporting this review are available from the authors on reasonable request and subject to publisher copyright restrictions.
