# Supplementary material for: Complement-amplified thrombotic microangiopathy in systemic lupus erythematosus and lupus nephritis: a systematic review of 78 reported patients
Source: Front Immunol. 2026 Sep 30;17:1940769. doi: 10.3389/fimmu.2026.1940769 (PMC13661993; doi:10.3389/fimmu.2026.1940769)
Supplement: Supplementary file 1 [file Table1.docx]

**Supplementary Material: SLE/LN-associated TMA systematic review (56 sources; 78 patients)**

Manuscript ID 1940769. Logic-aligned supplementary-table release: 23 August 2026. CNKI was not included; the registered-protocol deviation is disclosed. Published abstracts are retained as a lower-information stratum. NR = not reported; No = explicitly documented nonexposure/absence where applicable.

# Supplementary Table S1. Protocol deviations and amendment chronology

| **Date/stage** | **Element** | **Deviation/amendment** | **Handling** |
| --- | --- | --- | --- |
| 17 Mar 2026 | Identification search | Archived PubMed, Embase, and Web of Science exports created before registration | Disclosed; not prospective for identification stage |
| 29 May 2026 | PROSPERO start | Registration followed identification search | Chronology stated in Methods |
| Original workflow | CNKI | Listed in PROSPERO; no verifiable export/search history/screening log | Not claimed as zero-yield; not added in bounded revision; bias acknowledged |
| Original workflow | Screening/extraction/appraisal | Initially one reviewer rather than two independent reviewers | Material deviation disclosed |
| Revision | Source/data audit | Computationally assisted primary-source and patient-key checks | Mitigation only; not described as dual-human review |
| 18 Mar–14 Aug 2026 | Update | PubMed only | Not described as a common-date multi-database update |
| Revision | GRADE-informed assessment | Completed after omission in submitted version | All key domains very low/not estimable |
| Revision | Yu 2026 | Author-known report in other-methods lane | Authorship disclosed; leave-one-source-out sensitivity reported |

# Supplementary Table S2. Source crosswalk and traceability

| **Source ID** | **Source (year)** | **Level/type** | **Patients** | **Lane** | **Identifier(s)** | **Correction/provenance** | **Traceability/author relationship** |
| --- | --- | --- | --- | --- | --- | --- | --- |
| S001 | Al Shohaib 2000 (2000) | full_text; full_text_case_report_letter_or_brief | 1 | B: other methods | NR | database-export linkage corrected in revision | primary_full_text_reaudited; author relationship=no |
| S002 | Ogawa 2000 (2000) | full_text; full_text_case_report_letter_or_brief | 1 | B: other methods | NR | database-export linkage corrected in revision | primary_full_text_reaudited; author relationship=no |
| S003 | Kawasaki 2002 (2002) | full_text; full_text_case_report | 1 | A: archived databases | DOI 10.1159/000065286; PMID 12381963 | none | primary_full_text_reaudited; author relationship=no |
| S004 | Tsao 2002 (2002) | full_text; full_text_case_report | 1 | A: archived databases | DOI 10.1007/s00296-001-0153-z; PMID 11843173 | none | primary_full_text_reaudited; author relationship=no |
| S005 | Rabbani 2005 (2005) | full_text; full_text_case_report | 1 | A: archived databases | PMID 15813638 | none | primary_full_text_reaudited; author relationship=no |
| S006 | Hunt 2007 (2007) | full_text; full_text_case_series | 3 | B: other methods | NR | database-export linkage corrected in revision | primary_full_text_reaudited; author relationship=no |
| S007 | Leban 2011 (2011) | full_text; full_text_case_series | 1 | A: archived databases | DOI 10.1007/s11255-010-9754-3; PMID 20499172 | none | primary_full_text_reaudited; author relationship=no |
| S008 | Samson 2012 (2012) | full_text; full_text_case_report_letter_or_brief | 1 | A: archived databases | DOI 10.1111/j.1445-5994.2011.02591.x; PMID 22276560 | none | primary_full_text_reaudited; author relationship=no |
| S009 | Shapira 2012 (2012) | full_text; full_text_case_report_letter_or_brief | 1 | B: other methods | NR | database-export linkage corrected in revision | primary_full_text_reaudited; author relationship=no |
| S010 | Pierer 2013 (2013) | published_abstract; published_conference_abstract | 1 | A: archived databases | DOI 10.1007/s00393-013-1255-1 | none | published_abstract_reaudited; author relationship=no |
| S011 | Kronbichler 2014 (2014) | full_text; full_text_case_report | 1 | B: other methods | NR | database-export linkage corrected in revision | primary_full_text_reaudited; author relationship=no |
| S012 | Almaani 2015 (2015) | published_abstract; published_conference_abstract | 1 | A: archived databases | NR | none | published_abstract_reaudited; author relationship=no |
| S013 | Blum 2015 (2015) | full_text; full_text_case_report | 1 | A: archived databases | DOI 10.5527/wjn.v4.i5.528; PMID 26558190 | none | primary_full_text_reaudited; author relationship=no |
| S014 | Coppo 2015 (2015) | full_text; full_text_case_report_letter_or_brief | 1 | A: archived databases | DOI 10.1007/s00467-014-2944-y; PMID 25173358 | none | primary_full_text_reaudited; author relationship=no |
| S015 | El-Husseini 2015 (2015) | full_text; full_text_case_report | 1 | A: archived databases | DOI 10.1053/j.ajkd.2014.07.031; PMID 25446020 | none | primary_full_text_reaudited; author relationship=no |
| S017 | Bermea 2016 (2016) | full_text; full_text_case_series | 2 | A: archived databases | DOI 10.1097/rhu.0000000000000423; PMID 27556240 | none | primary_full_text_reaudited; author relationship=no |
| S018 | Raufi 2016 (2016) | full_text; full_text_case_report | 1 | A: archived databases | DOI 10.4081/hr.2016.6625 | none | primary_full_text_reaudited; author relationship=no |
| S019 | Sarkissian 2016 (2016) | published_abstract; published_conference_abstract | 1 | A: archived databases | DOI 10.1136/jim-d-15-00013.191 | none | published_abstract_reaudited; author relationship=no |
| S020 | Taktak 2016 (2016) | full_text; full_text_case_report | 1 | A: archived databases | DOI 10.24953/turkjped.2016.02.018; PMID 27976568 | none | primary_full_text_reaudited; author relationship=no |
| S021 | Wig 2016 (2016) | full_text; full_text_case_report_letter_or_brief | 1 | B: other methods | NR | database-export linkage corrected in revision | primary_full_text_reaudited; author relationship=no |
| S022 | Huzair Ali 2017 (2017) | published_abstract; published_conference_abstract | 1 | A: archived databases | NR | none | published_abstract_reaudited; author relationship=no |
| S023 | de Holanda 2017 (2017) | full_text; full_text_case_report | 1 | A: archived databases | DOI 10.1007/s10067-017-3823-2; PMID 28905254 | none | primary_full_text_reaudited; author relationship=no |
| S024 | Attar 2018 (2018) | full_text; full_text_case_report | 1 | B: other methods | NR | database-export linkage corrected in revision | primary_full_text_reaudited; author relationship=no |
| S025 | Bangaru 2018 (2018) | published_abstract; published_conference_abstract | 1 | A: archived databases | NR | none | published_abstract_reaudited; author relationship=no |
| S026 | Gupta 2018 (2018) | published_abstract; published_conference_abstract | 1 | A: archived databases | NR | none | published_abstract_reaudited; author relationship=no |
| S027 | Ono 2018 (2018) | full_text; full_text_case_report | 1 | A: archived databases | DOI 10.2169/internalmedicine.0228-17; PMID 29434134 | none | primary_full_text_reaudited; author relationship=no |
| S028 | Park 2018 (2018) | full_text; full_text_case_series | 11 | A: archived databases | DOI 10.1182/bloodadvances.2018019596; PMID 30131343 | none | primary_full_text_reaudited; author relationship=no |
| S029 | Syed 2018 (2018) | published_abstract; published_conference_abstract | 1 | A: archived databases | NR | none | published_abstract_reaudited; author relationship=no |
| S030 | Kello 2019 (2019) | full_text; full_text_case_series | 9 | A: archived databases | DOI 10.1016/j.semarthrit.2018.11.005; PMID 30598332 | none | primary_full_text_reaudited; author relationship=no |
| S031 | Skoczyń-ska 2019 (2019) | published_abstract; published_conference_abstract | 1 | A: archived databases | NR | none | published_abstract_reaudited; author relationship=no |
| S032 | Drexler 2021 (2021) | published_abstract; published_conference_abstract | 1 | A: archived databases | NR | none | published_abstract_reaudited; author relationship=no |
| S033 | Kim 2021 (2021) | full_text; full_text_case_report | 1 | A: archived databases | DOI 10.1186/s12882-021-02293-2; PMID 33691638 | none | primary_full_text_reaudited; author relationship=no |
| S034 | Su 2021 (2021) | full_text; full_text_case_series | 2 | A: archived databases | DOI 10.1093/rheumatology/keaa474; PMID 32893307 | none | primary_full_text_reaudited; author relationship=no |
| S035 | Torres 2021 (2021) | full_text; full_text_case_report | 1 | A: archived databases | DOI 10.1159/000512227 | none | primary_full_text_reaudited; author relationship=no |
| S036 | Xie 2021 (2021) | published_abstract; published_conference_abstract | 1 | A: archived databases | DOI 10.1159/000515882 | none | published_abstract_reaudited; author relationship=no |
| S037 | AlGhobaishi 2022 (2022) | full_text; full_text_case_report | 1 | A: archived databases | DOI 10.1016/j.amsu.2022.104541 | none | primary_full_text_reaudited; author relationship=no |
| S038 | Alhamoud 2022 (2022) | full_text; full_text_case_report | 1 | A: archived databases | DOI 10.7759/cureus.25117; PMID 35733457 | none | primary_full_text_reaudited; author relationship=no |
| S039 | Amin 2023 (2023) | published_abstract; published_conference_abstract | 1 | A: archived databases | DOI 10.1681/asn.20233411s1277c | none | published_abstract_reaudited; author relationship=no |
| S040 | Brill 2023 (2023) | published_abstract; published_conference_abstract | 1 | A: archived databases | DOI 10.1016/j.chest.2023.07.3613 | none | published_abstract_reaudited; author relationship=no |
| S041 | Lin 2023 (2023) | published_abstract; published_conference_abstract | 1 | A: archived databases | DOI 10.1007/s11606-023-08226-z | none | published_abstract_reaudited; author relationship=no |
| S042 | Díaz Góngora 2024 (2024) | published_abstract; published_conference_abstract | 1 | A: archived databases | DOI 10.1016/j.ekir.2024.02.1089 | none | published_abstract_reaudited; author relationship=no |
| S043 | Smith 2024 (2024) | full_text; full_text_case_report | 1 | A: archived databases | DOI 10.1007/s00296-024-05558-9; PMID 38502235 | none | primary_full_text_reaudited; author relationship=no |
| S044 | Benvenuti 2025 (2025) | full_text; full_text_case_report | 1 | A: archived databases | DOI 10.1007/s40620-025-02425-z; PMID 40996634 | none | primary_full_text_reaudited; author relationship=no |
| S045 | Inatomi 2025 (2025) | full_text; full_text_case_report | 1 | A: archived databases | DOI 10.7759/cureus.78989; PMID 40092016 | none | primary_full_text_reaudited; author relationship=no |
| S046 | Thomson 2025 (2025) | full_text; full_text_case_report | 1 | A: archived databases | DOI 10.1136/bcr-2025-266485; PMID 41173493 | none | primary_full_text_reaudited; author relationship=no |
| S047 | Yu 2026 (2026) | full_text; full_text_case_report | 1 | B: other methods | DOI 10.1093/qjmed/hcag151; PMID 42475512 | manual author-known inclusion; update-search duplicate removed | primary_full_text_reaudited; author relationship=yes |
| S048 | Sun 2026 (2026) | full_text; full_text_case_series | 1 | C: PubMed update | DOI 10.3389/fphar.2026.1791188; PMID 42591843 | new report from PubMed-only update | primary_full_text_reaudited; author relationship=no |
| S049 | Pokharel 2026 (2026) | full_text; full_text_case_report | 1 | C: PubMed update | DOI 10.7759/cureus.111765; PMID 42534175 | new report from PubMed-only update | primary_full_text_reaudited; author relationship=no |
| S050 | Lin 2026 (2026) | full_text; full_text_case_report | 1 | C: PubMed update | DOI 10.1097/md.0000000000046412; PMID 42470044 | new report from PubMed-only update | primary_full_text_reaudited; author relationship=no |
| S051 | Nickavar 2026 (2026) | full_text; full_text_case_report | 1 | C: PubMed update | DOI 10.61882/ijkd.3.03.9466; PMID 42234925 | new report from PubMed-only update | primary_full_text_reaudited; author relationship=no |
| S052 | Stirnkorb 2026 (2026) | published_abstract; published_journal_article_abstract_only | 1 | B: other methods | DOI 10.1007/s00467-025-07108-2; PMID 41838093 | search/export omission relative to archived files | published_abstract_reaudited; author relationship=no |
| S053 | Fukunaga 2026 (2026) | full_text; full_text_case_report | 1 | A: archived databases | DOI 10.7759/cureus.100622; PMID 41635378 | screening omission corrected | primary_full_text_reaudited; author relationship=no |
| S054 | Baltaci 2026 (2026) | full_text; full_text_case_report | 1 | B: other methods | DOI 10.1177/15563316241300474; PMID 39605987 | search/export omission relative to archived files | primary_full_text_reaudited; author relationship=no |
| S055 | Kaneda 2020 (2020) | full_text; full_text_case_report | 1 | B: other methods | DOI 10.1007/s13730-020-00475-9; PMID 32304042 | search/export omission relative to archived files | primary_full_text_reaudited; author relationship=no |
| S056 | Khan 2020 (2020) | published_abstract; published_conference_abstract | 1 | A: archived databases | DOI 10.1016/j.chest.2020.08.725 | wrong-attachment exclusion corrected | published_abstract_reaudited; author relationship=no |
| S057 | Theodoru 2023 (2023) | published_abstract; published_conference_abstract | 1 | A: archived databases | DOI 10.1681/asn.20233411s1951b | wrong-attachment exclusion corrected | published_abstract_reaudited; author relationship=no |

# Supplementary Table S3. Screening and source-integrity decisions

| **Lane** | **Record/stage** | **Decision/count** | **Reason/handling** |
| --- | --- | --- | --- |
| Archived databases | PubMed/Embase/WOS exports | 598 identified; 105 duplicates; 493 screened | 451 excluded; 42 reports assessed |
| Archived databases | Patel 2025 | Exclude | Index allograft C3GN; no current clinical or biopsy TMA |
| Archived databases | Final archived lane | Include 41 reports | Database-linked reports retained after source audit |
| Other methods | Seven legacy manual additions | Include | Not linkable to the retained 493-record export; provenance explicitly reassigned |
| Other methods | Stirnkorb 2026; Baltaci 2026; Kaneda 2020 | Include | Eligible pre-cutoff reports recovered by targeted omission audit |
| Other methods | Yu 2026 | Include | Author-known accepted report; later update duplicate removed; relationship disclosed |
| Other methods | Pickering 2015 | Exclude | Primary report explicitly described non-TMA glomerular lesions |
| PubMed update | 18 Mar–14 Aug 2026 | 19 records; one prior-manual duplicate; 18 screened | 11 excluded; 7 assessed; 3 aggregate-only excluded; 4 included |
| Final synthesis | All lanes | 56 sources; 78 patients | 39 full-text sources/61 patients; 17 published abstracts/17 patients |
| Registered deviation | CNKI | Not searched/verified in the archived workflow and not added in this bounded revision | Potential database and language bias acknowledged |

# Supplementary Table S4. Key data definitions and reproducibility record

| **Field/domain** | **Definition** | **Use/interpretation** |
| --- | --- | --- |
| patient_id/source_id | Stable patient/source keys; source IDs are not renumbered after exclusion | Traceability and joins |
| Phenotype states | present / absent / not_reported / unclear | Figure 8 and patient matrix; missing is not negative |
| Strict T2 | P/LP complement finding or positive anti-factor H | Primary complement evidence analysis |
| Extended T2 | Strict T2 plus source-described deleterious/author-causal finding without formal P/LP | Sensitivity analysis only |
| T3 | Index-event terminal-pathway biomarker or tissue complement-activation signal | Proximal mechanistic evidence |
| Treatment exposure | Documented administration; any-time and index-episode fields separated | Not reported is not nonuse |
| Index inhibitor class | No inhibitor / eculizumab only / other complement sequence | Mutually exclusive Figure 3 grouping |
| Renal outcome | Improved/recovered / kidney-function loss / death-no renal recovery / unclear | Descriptive outcome harmonization |
| Hematologic outcome | Improved / no response / unclear | Descriptive outcome harmonization |
| AI boundary | Language/code/document assistance; generative visual concept/redrawing assistance for schematic Figures 1–2; deterministic conventional-code visualization for Figure 8 | Authors corrected and verified all labels and numerical content and accept responsibility |

## Release manifest

| **File** | **Bytes** | **SHA-256** |
| --- | --- | --- |
| minimal_revision78_analysis_ready.csv | 575584 | ba2a11fde56d9eaf18de4df13382b4038c4718f427115ab904c437f4810a6e5a |
| minimal_revision56_source_crosswalk.csv | 68182 | 68826f9cc3df2185ab88dc5b3319131f63dc34ba04c4ce76334e3f75beca4ce4 |
| minimal_revision78_summary.json | 13605 | 02f9d899f562152b716f0740d5d28bc37060fb51ed7ab0676b7f10dc4258ded2 |
| minimal_revision78_qc.json | 1214 | 05f77221fab06c971fbbe4a511198dd7824487276ab2621d083d1fcf98ad8e9e |

# Supplementary Table S5. Patient-level extraction matrix

| **No.** | **Patient** | **Source; case** | **Level** | **Country** | **Sex; age** | **SLE/LN/pathology** | **Phenotype states** | **Contexts** | **ADAMTS13** | **Complement evidence** | **Other treatments** | **Complement agent/index class** | **Renal outcome** | **Hematologic outcome** | **Follow-up** |
| --- | --- | --- | --- | --- | --- | --- | --- | --- | --- | --- | --- | --- | --- | --- | --- |
| 1 | P001 | Al Shohaib 2000; case 1 | full_text | Saudi Arabia | F; 18.0 y | Class IV / diffuse proliferative LN | MAHA=present; PLT=present; AKI=present; biopsy-TMA=not_reported; RRT=not_reported | none clearly reported | NR | T1=not documented; strict T2=not documented; T3=not documented; VUS/uncertain=not documented; negative assay=not documented | PLEX=Y; CYC=NR; MMF=NR; RTX=NR; IVIG=NR; CNI=NR; AC=NR; AP=NR | none reported; index=No complement inhibitor | Improved/recovered | Improved | Improved/recovered or dialysis-independent; short-term follow-up |
| 2 | P002 | Ogawa 2000; case 1 | full_text | Japan | F; 10.0 y | Class II / mesangial LN | MAHA=present; PLT=present; AKI=present; biopsy-TMA=absent; RRT=not_reported | DAH/lung involvement; neurologic involvement | NR | T1=Y; strict T2=not documented; T3=not documented; VUS/uncertain=not documented; negative assay=not documented | PLEX=Y; CYC=Y; MMF=NR; RTX=NR; IVIG=NR; CNI=NR; AC=NR; AP=NR | none reported; index=No complement inhibitor | Improved/recovered | Improved | Improved/recovered or dialysis-independent; hematologic response |
| 3 | P003 | Kawasaki 2002; case 1 | full_text | Japan | F; 12.0 y | Class III / focal proliferative LN | MAHA=present; PLT=present; AKI=present; biopsy-TMA=absent; RRT=not_reported | none clearly reported | NR | T1=Y; strict T2=not documented; T3=not documented; VUS/uncertain=not documented; negative assay=not documented | PLEX=NR; CYC=NR; MMF=NR; RTX=NR; IVIG=NR; CNI=NR; AC=Y; AP=Y | none reported; index=No complement inhibitor | Improved/recovered | Improved | Improved/recovered or dialysis-independent; hematologic response; follow-up 1 years |
| 4 | P004 | Tsao 2002; case 1 | full_text | China | M; 15.0 y | Class IV / diffuse proliferative LN | MAHA=present; PLT=present; AKI=present; biopsy-TMA=present; RRT=not_reported | none clearly reported | NR | T1=Y; strict T2=not documented; T3=not documented; VUS/uncertain=not documented; negative assay=not documented | PLEX=No; CYC=Y; MMF=NR; RTX=NR; IVIG=NR; CNI=NR; AC=NR; AP=NR | none reported; index=No complement inhibitor | Improved/recovered | Improved | Improved/recovered or dialysis-independent; hematologic response; follow-up 3 months |
| 5 | P005 | Rabbani 2005; case 1 | full_text | Pakistan | F; 21.0 y | Class IV / diffuse proliferative LN | MAHA=present; PLT=present; AKI=present; biopsy-TMA=present; RRT=present | none clearly reported | NR | T1=Y; strict T2=not documented; T3=not documented; VUS/uncertain=not documented; negative assay=not documented | PLEX=Y; CYC=Y; MMF=NR; RTX=NR; IVIG=NR; CNI=NR; AC=NR; AP=NR | none reported; index=No complement inhibitor | Improved/recovered | Improved | Improved/recovered or dialysis-independent; hematologic response; lost to follow-up |
| 6 | P006 | Hunt 2007; case 1 | full_text | United Kingdom | F; 24.0 y | Class IV / diffuse proliferative LN | MAHA=present; PLT=present; AKI=present; biopsy-TMA=not_reported; RRT=not_reported | none clearly reported | vWF-CP 96%（normal） | T1=Y; strict T2=not documented; T3=not documented; VUS/uncertain=not documented; negative assay=not documented | PLEX=Y; CYC=Y; MMF=Y; RTX=Y; IVIG=Y; CNI=NR; AC=NR; AP=NR | none reported; index=No complement inhibitor | Improved/recovered | Improved | Improved/recovered or dialysis-independent; follow-up 6 weeks |
| 7 | P007 | Hunt 2007; case 2 | full_text | United Kingdom | F; 21.0 y | Class IV / diffuse proliferative LN | MAHA=present; PLT=present; AKI=present; biopsy-TMA=not_reported; RRT=present | neurologic involvement | vWF-CP 86%（normal） | T1=not documented; strict T2=not documented; T3=not documented; VUS/uncertain=not documented; negative assay=not documented | PLEX=Y; CYC=Y; MMF=Y; RTX=NR; IVIG=NR; CNI=NR; AC=NR; AP=NR | none reported; index=No complement inhibitor | Improved/recovered | Improved | Improved/recovered or dialysis-independent; hematologic response |
| 8 | P008 | Hunt 2007; case 3 | full_text | United Kingdom | F; 22.0 y | LN, class not specified | MAHA=present; PLT=present; AKI=present; biopsy-TMA=absent; RRT=not_reported | neurologic involvement | not reported; no severe deficiency stated | T1=not documented; strict T2=not documented; T3=not documented; VUS/uncertain=not documented; negative assay=not documented | PLEX=Y; CYC=Y; MMF=Y; RTX=NR; IVIG=Y; CNI=NR; AC=NR; AP=NR | none reported; index=No complement inhibitor | Improved/recovered | Improved | Partial or mixed outcome; hematologic response; short-term follow-up |
| 9 | P009 | Leban 2011; case 4 | full_text | Tunisia | F; 37.0 y | Class IV / diffuse proliferative LN | MAHA=unclear; PLT=present; AKI=absent; biopsy-TMA=present; RRT=absent | none clearly reported | NR | T1=Y; strict T2=not documented; T3=not documented; VUS/uncertain=not documented; negative assay=Y | PLEX=NR; CYC=Y; MMF=NR; RTX=NR; IVIG=NR; CNI=NR; AC=NR; AP=NR | none reported; index=No complement inhibitor | Unclear | Improved | Kidney function loss / dialysis-dependent; hematologic response; short-term follow-up |
| 10 | P010 | Samson 2012; case 1 | full_text | France | F; 32.0 y | Class IV / diffuse proliferative LN | MAHA=present; PLT=present; AKI=present; biopsy-TMA=absent; RRT=not_reported | none clearly reported | 33%, mildly reduced | T1=Y; strict T2=not documented; T3=not documented; VUS/uncertain=not documented; negative assay=not documented | PLEX=Y; CYC=Y; MMF=Y; RTX=NR; IVIG=NR; CNI=NR; AC=NR; AP=NR | none reported; index=No complement inhibitor | Improved/recovered | Improved | Improved/recovered or dialysis-independent; hematologic response; follow-up 15 months; no recurrence reported |
| 11 | P011 | Shapira 2012; case 1 | full_text | United States | M; 28.0 y | LN, class not specified | MAHA=present; PLT=present; AKI=not_reported; biopsy-TMA=not_reported; RRT=not_reported | APS/CAPS/aPL | NR | T1=not documented; strict T2=not documented; T3=not documented; VUS/uncertain=not documented; negative assay=not documented | PLEX=Y; CYC=Y; MMF=NR; RTX=Y; IVIG=Y; CNI=NR; AC=Y; AP=Y | eculizumab; index=Eculizumab only | Unclear | Improved | Unclear/not reported; follow-up 3 years |
| 12 | P012 | Pierer 2013; case 1 | published_abstract | Germany | F; NR y | LN, class not specified | MAHA=present; PLT=present; AKI=present; biopsy-TMA=present; RRT=present | neurologic involvement | NR | T1=not documented; strict T2=not documented; T3=not documented; VUS/uncertain=not documented; negative assay=Y | PLEX=Y; CYC=NR; MMF=NR; RTX=NR; IVIG=NR; CNI=NR; AC=NR; AP=NR | eculizumab; index=Eculizumab only | Improved/recovered | Improved | Improved/recovered or dialysis-independent; hematologic response; remission reported |
| 13 | P013 | Kronbichler 2014; case 1 | full_text | Austria | F; 30.0 y | LN, class not specified | MAHA=present; PLT=present; AKI=present; biopsy-TMA=present; RRT=present | pregnancy/postpartum; APS/CAPS/aPL | NR | T1=Y; strict T2=not documented; T3=not documented; VUS/uncertain=Y; negative assay=Y | PLEX=Y; CYC=NR; MMF=Y; RTX=Y; IVIG=No; CNI=NR; AC=Y; AP=NR | eculizumab; index=Eculizumab only | Kidney function loss | Improved | Kidney function loss / dialysis-dependent; hematologic response; follow-up 3 months |
| 14 | P014 | Almaani 2015; case 1 | published_abstract | United States | F; 21.0 y | Class II / mesangial LN | MAHA=present; PLT=present; AKI=present; biopsy-TMA=present; RRT=present | APS/CAPS/aPL | normal | T1=not documented; strict T2=not documented; T3=not documented; VUS/uncertain=not documented; negative assay=not documented | PLEX=Y; CYC=NR; MMF=NR; RTX=NR; IVIG=NR; CNI=NR; AC=NR; AP=NR | eculizumab; index=Eculizumab only | Improved/recovered | Improved | Improved/recovered or dialysis-independent; hematologic response; remission reported |
| 15 | P015 | Blum 2015; case 1 | full_text | Canada | F; 44.0 y | Class IV / diffuse proliferative LN | MAHA=present; PLT=present; AKI=present; biopsy-TMA=absent; RRT=present | none clearly reported | 0.49 | T1=Y; strict T2=not documented; T3=not documented; VUS/uncertain=not documented; negative assay=not documented | PLEX=Y; CYC=Y; MMF=Y; RTX=NR; IVIG=NR; CNI=NR; AC=NR; AP=NR | none reported; index=No complement inhibitor | Improved/recovered | Improved | Partial or mixed outcome; hematologic response; remission reported |
| 16 | P016 | Coppo 2015; case 1 | full_text | Italy | F; 4.0 y | Class IV / diffuse proliferative LN | MAHA=present; PLT=present; AKI=present; biopsy-TMA=absent; RRT=not_reported | none clearly reported | normal; anti-ADAMTS13 antibody negative | T1=Y; strict T2=not documented; T3=Y; VUS/uncertain=not documented; negative assay=Y | PLEX=Y; CYC=Y; MMF=Y; RTX=Y; IVIG=NR; CNI=Y; AC=NR; AP=NR | eculizumab; index=Eculizumab only | Improved/recovered | Improved | Improved/recovered or dialysis-independent; hematologic response; follow-up 17 months |
| 17 | P017 | El-Husseini 2015; case 1 | full_text | United States | F; 24.0 y | Mixed proliferative + membranous LN | MAHA=present; PLT=present; AKI=present; biopsy-TMA=present; RRT=present | none clearly reported | below normal but not consistent with classic TTP | T1=Y; strict T2=not documented; T3=not documented; VUS/uncertain=not documented; negative assay=Y | PLEX=Y; CYC=Y; MMF=Y; RTX=NR; IVIG=NR; CNI=NR; AC=NR; AP=NR | eculizumab; index=Eculizumab only | Improved/recovered | Improved | Improved/recovered or dialysis-independent; hematologic response; follow-up 1 years; remission reported |
| 18 | P019 | Bermea 2016; case 1 | full_text | United States | F; 30.0 y | Class III / focal proliferative LN | MAHA=present; PLT=present; AKI=present; biopsy-TMA=absent; RRT=present | none clearly reported | 0.71 | T1=Y; strict T2=not documented; T3=not documented; VUS/uncertain=not documented; negative assay=not documented | PLEX=Y; CYC=Y; MMF=NR; RTX=Y; IVIG=Y; CNI=NR; AC=NR; AP=NR | eculizumab; index=Eculizumab only | Kidney function loss | Improved | Kidney function loss / dialysis-dependent; hematologic response; follow-up 1 years; remission reported |
| 19 | P020 | Bermea 2016; case 2 | full_text | United States | M; 21.0 y | Class II / mesangial LN | MAHA=present; PLT=present; AKI=present; biopsy-TMA=absent; RRT=present | DAH/lung involvement | NR / not severe deficiency | T1=Y; strict T2=not documented; T3=not documented; VUS/uncertain=not documented; negative assay=not documented | PLEX=Y; CYC=Y; MMF=Y; RTX=Y; IVIG=Y; CNI=NR; AC=NR; AP=NR | eculizumab; index=Eculizumab only | Kidney function loss | Improved | Kidney function loss / dialysis-dependent; hematologic response; follow-up 14 months |
| 20 | P021 | Raufi 2016; case 1 | full_text | United States | F; 25.0 y | Class IV / diffuse proliferative LN | MAHA=present; PLT=present; AKI=present; biopsy-TMA=present; RRT=not_reported | infection | 0.99 | T1=Y; strict T2=not documented; T3=not documented; VUS/uncertain=not documented; negative assay=Y | PLEX=Y; CYC=Y; MMF=Y; RTX=Y; IVIG=NR; CNI=NR; AC=NR; AP=NR | eculizumab; index=Eculizumab only | Improved/recovered | Improved | Improved/recovered or dialysis-independent; hematologic response; remission reported |
| 21 | P022 | Sarkissian 2016; case 1 | published_abstract | United States | F; 25.0 y | Class IV / diffuse proliferative LN | MAHA=present; PLT=present; AKI=present; biopsy-TMA=present; RRT=not_reported | none clearly reported | 0.99 | T1=Y; strict T2=not documented; T3=not documented; VUS/uncertain=not documented; negative assay=not documented | PLEX=Y; CYC=Y; MMF=NR; RTX=Y; IVIG=NR; CNI=NR; AC=NR; AP=NR | eculizumab; index=Eculizumab only | Improved/recovered | Improved | Improved/recovered or dialysis-independent; hematologic response |
| 22 | P023 | Taktak 2016; case 1 | full_text | Türkiye | M; 15.0 y | Class II / mesangial LN | MAHA=present; PLT=present; AKI=present; biopsy-TMA=present; RRT=not_reported | none clearly reported | 0.98 | T1=Y; strict T2=not documented; T3=not documented; VUS/uncertain=not documented; negative assay=not documented | PLEX=NR; CYC=NR; MMF=NR; RTX=NR; IVIG=NR; CNI=NR; AC=NR; AP=NR | none reported; index=No complement inhibitor | Improved/recovered | Improved | Improved/recovered or dialysis-independent; no recurrence reported |
| 23 | P024 | Wig 2016; case 1 | full_text | United Kingdom | F; 43.0 y | LN, class not specified | MAHA=not_reported; PLT=present; AKI=present; biopsy-TMA=present; RRT=absent | APS/CAPS/aPL | normal | T1=not documented; strict T2=not documented; T3=not documented; VUS/uncertain=not documented; negative assay=not documented | PLEX=Y; CYC=Y; MMF=Y; RTX=Y; IVIG=Y; CNI=NR; AC=Y; AP=Y | eculizumab; index=Eculizumab only | Improved/recovered | Improved | Partial or mixed outcome |
| 24 | P025 | Huzair Ali 2017; case 1 | published_abstract | United States | M; 19.0 y | Class IV / diffuse proliferative LN | MAHA=present; PLT=present; AKI=present; biopsy-TMA=present; RRT=present | infection | normal | T1=Y; strict T2=not documented; T3=not documented; VUS/uncertain=Y; negative assay=not documented | PLEX=Y; CYC=NR; MMF=Y; RTX=Y; IVIG=NR; CNI=NR; AC=NR; AP=NR | eculizumab; index=Eculizumab only | Improved/recovered | Improved | Improved/recovered or dialysis-independent; hematologic response; discharged |
| 25 | P026 | de Holanda 2017; case 1 | full_text | Brazil | F; 18.0 y | Class IV / diffuse proliferative LN | MAHA=present; PLT=present; AKI=present; biopsy-TMA=present; RRT=present | none clearly reported | 0.58 | T1=Y; strict T2=not documented; T3=not documented; VUS/uncertain=Y; negative assay=not documented | PLEX=Y; CYC=Y; MMF=NR; RTX=NR; IVIG=NR; CNI=NR; AC=NR; AP=NR | eculizumab; index=Eculizumab only | Improved/recovered | Improved | Improved/recovered or dialysis-independent; follow-up 3 years |
| 26 | P027 | Attar 2018; case 1 | full_text | Saudi Arabia | M; 9.0 y | LN, class not specified | MAHA=present; PLT=present; AKI=present; biopsy-TMA=present; RRT=present | none clearly reported | NR | T1=Y; strict T2=not documented; T3=not documented; VUS/uncertain=not documented; negative assay=not documented | PLEX=No; CYC=Y; MMF=NR; RTX=NR; IVIG=NR; CNI=NR; AC=NR; AP=NR | none reported; index=No complement inhibitor | Improved/recovered | Improved | Improved/recovered or dialysis-independent; hematologic response |
| 27 | P028 | Bangaru 2018; case 1 | published_abstract | United States | F; 20.0 y | Class III / focal proliferative LN | MAHA=present; PLT=present; AKI=present; biopsy-TMA=present; RRT=present | pregnancy/postpartum | 1 | T1=not documented; strict T2=not documented; T3=not documented; VUS/uncertain=not documented; negative assay=not documented | PLEX=Y; CYC=Y; MMF=Y; RTX=NR; IVIG=NR; CNI=NR; AC=NR; AP=NR | eculizumab; index=Eculizumab only | Kidney function loss | Unclear | Partial or mixed outcome; hematologic response |
| 28 | P029 | Gupta 2018; case 1 | published_abstract | United States | M; 20.0 y | Mixed proliferative + membranous LN | MAHA=present; PLT=present; AKI=present; biopsy-TMA=present; RRT=present | DAH/lung involvement | 0.86 | T1=Y; strict T2=not documented; T3=not documented; VUS/uncertain=not documented; negative assay=not documented | PLEX=Y; CYC=Y; MMF=NR; RTX=NR; IVIG=NR; CNI=NR; AC=NR; AP=NR | eculizumab; index=Eculizumab only | Death/no renal recovery | Improved | Kidney function loss / dialysis-dependent; hematologic response |
| 29 | P030 | Ono 2018; case 1 | full_text | Japan | F; 31.0 y | Class V / membranous LN | MAHA=present; PLT=present; AKI=present; biopsy-TMA=present; RRT=present | none clearly reported | 83%, not reduced | T1=Y; strict T2=not documented; T3=not documented; VUS/uncertain=not documented; negative assay=Y | PLEX=Y; CYC=NR; MMF=NR; RTX=NR; IVIG=NR; CNI=NR; AC=NR; AP=NR | eculizumab; index=Eculizumab only | Kidney function loss | Improved | Partial or mixed outcome; hematologic response |
| 30 | P031 | Park 2018; case 1 | full_text | United States | F; 39.0 y | Class V / membranous LN | MAHA=present; PLT=present; AKI=present; biopsy-TMA=present; RRT=present | none clearly reported | NR | T1=not documented; strict T2=not documented; T3=not documented; VUS/uncertain=not documented; negative assay=Y | PLEX=Y; CYC=Y; MMF=NR; RTX=NR; IVIG=NR; CNI=NR; AC=NR; AP=NR | eculizumab; index=Eculizumab only | Kidney function loss | Improved | Partial or mixed outcome; follow-up 190 weeks |
| 31 | P032 | Park 2018; case 10 | full_text | United States | F; 34.0 y | Unclear or non-LN dominant | MAHA=present; PLT=present; AKI=present; biopsy-TMA=present; RRT=present | APS/CAPS/aPL | 1.51 | T1=not documented; strict T2=not documented; T3=not documented; VUS/uncertain=not documented; negative assay=Y | PLEX=Y; CYC=Y; MMF=NR; RTX=NR; IVIG=NR; CNI=NR; AC=Y; AP=NR | eculizumab; index=Eculizumab only | Improved/recovered | Improved | Improved/recovered or dialysis-independent; death reported |
| 32 | P033 | Park 2018; case 11 | full_text | United States | M; 57.0 y | Unclear or non-LN dominant | MAHA=present; PLT=present; AKI=present; biopsy-TMA=not_reported; RRT=present | APS/CAPS/aPL | NR | T1=not documented; strict T2=not documented; T3=not documented; VUS/uncertain=Y; negative assay=Y | PLEX=Y; CYC=Y; MMF=NR; RTX=NR; IVIG=NR; CNI=NR; AC=Y; AP=NR | eculizumab; index=Eculizumab only | Improved/recovered | Unclear | Improved/recovered or dialysis-independent; follow-up 7 weeks |
| 33 | P034 | Park 2018; case 2 | full_text | United States | F; 25.0 y | Class IV / diffuse proliferative LN | MAHA=present; PLT=present; AKI=present; biopsy-TMA=present; RRT=present | none clearly reported | 0.67 | T1=Y; strict T2=not documented; T3=not documented; VUS/uncertain=Y; negative assay=Y | PLEX=Y; CYC=Y; MMF=NR; RTX=NR; IVIG=NR; CNI=NR; AC=NR; AP=NR | eculizumab; index=Eculizumab only | Improved/recovered | Improved | Improved/recovered or dialysis-independent; follow-up 139 weeks |
| 34 | P035 | Park 2018; case 3 | full_text | United States | F; 22.0 y | Class IV / diffuse proliferative LN | MAHA=present; PLT=present; AKI=present; biopsy-TMA=present; RRT=present | none clearly reported | 0.98 | T1=Y; strict T2=not documented; T3=not documented; VUS/uncertain=not documented; negative assay=Y | PLEX=Y; CYC=Y; MMF=NR; RTX=NR; IVIG=NR; CNI=NR; AC=NR; AP=NR | eculizumab; index=Eculizumab only | Improved/recovered | Improved | Improved/recovered or dialysis-independent; follow-up 124 weeks |
| 35 | P036 | Park 2018; case 4 | full_text | United States | F; 37.0 y | LN, class not specified | MAHA=present; PLT=present; AKI=present; biopsy-TMA=not_reported; RRT=present | none clearly reported | NR | T1=Y; strict T2=not documented; T3=not documented; VUS/uncertain=not documented; negative assay=Y | PLEX=Y; CYC=Y; MMF=NR; RTX=NR; IVIG=NR; CNI=NR; AC=NR; AP=NR | eculizumab; index=Eculizumab only | Kidney function loss | Improved | Partial or mixed outcome; follow-up 170 weeks |
| 36 | P037 | Park 2018; case 5 | full_text | United States | F; 31.0 y | Class III / focal proliferative LN | MAHA=present; PLT=present; AKI=present; biopsy-TMA=present; RRT=not_reported | none clearly reported | 0.89 | T1=Y; strict T2=not documented; T3=not documented; VUS/uncertain=not documented; negative assay=not documented | PLEX=Y; CYC=Y; MMF=NR; RTX=NR; IVIG=NR; CNI=NR; AC=NR; AP=NR | eculizumab; index=Eculizumab only | Death/no renal recovery | No response | Kidney function loss / dialysis-dependent; death reported |
| 37 | P038 | Park 2018; case 6 | full_text | United States | F; 42.0 y | Unclear or non-LN dominant | MAHA=present; PLT=present; AKI=present; biopsy-TMA=present; RRT=absent | APS/CAPS/aPL | 1.23 | T1=Y; strict T2=not documented; T3=not documented; VUS/uncertain=Y; negative assay=Y | PLEX=Y; CYC=Y; MMF=NR; RTX=NR; IVIG=NR; CNI=NR; AC=Y; AP=NR | eculizumab; index=Eculizumab only | Improved/recovered | Improved | Improved/recovered or dialysis-independent; follow-up 75 weeks |
| 38 | P039 | Park 2018; case 7 | full_text | United States | F; 59.0 y | LN, class not specified | MAHA=present; PLT=present; AKI=present; biopsy-TMA=not_reported; RRT=absent | none clearly reported | 0.99 | T1=Y; strict T2=not documented; T3=not documented; VUS/uncertain=not documented; negative assay=Y | PLEX=Y; CYC=Y; MMF=NR; RTX=NR; IVIG=NR; CNI=NR; AC=NR; AP=NR | eculizumab; index=Eculizumab only | Improved/recovered | Improved | Improved/recovered or dialysis-independent; follow-up 52 weeks; no recurrence reported |
| 39 | P040 | Park 2018; case 8 | full_text | United States | F; 22.0 y | Mixed proliferative + membranous LN | MAHA=present; PLT=present; AKI=present; biopsy-TMA=absent; RRT=present | none clearly reported | NR | T1=Y; strict T2=not documented; T3=not documented; VUS/uncertain=Y; negative assay=Y | PLEX=Y; CYC=Y; MMF=NR; RTX=NR; IVIG=NR; CNI=NR; AC=Y; AP=NR | eculizumab; index=Eculizumab only | Kidney function loss | Improved | Improved/recovered or dialysis-independent; follow-up 26 weeks |
| 40 | P041 | Park 2018; case 9 | full_text | United States | M; 31.0 y | Class IV / diffuse proliferative LN | MAHA=present; PLT=present; AKI=present; biopsy-TMA=present; RRT=present | none clearly reported | 0.89 | T1=not documented; strict T2=not documented; T3=not documented; VUS/uncertain=Y; negative assay=Y | PLEX=Y; CYC=Y; MMF=NR; RTX=NR; IVIG=NR; CNI=NR; AC=NR; AP=NR | eculizumab; index=Eculizumab only | Improved/recovered | Unclear | Partial or mixed outcome; follow-up 36 weeks |
| 41 | P042 | Syed 2018; case 1 | published_abstract | United States | F; 25.0 y | Class III / focal proliferative LN | MAHA=present; PLT=present; AKI=present; biopsy-TMA=present; RRT=present | neurologic involvement | TTP reportedly excluded; exact value not reported | T1=not documented; strict T2=not documented; T3=Y; VUS/uncertain=not documented; negative assay=Y | PLEX=No; CYC=Y; MMF=Y; RTX=NR; IVIG=NR; CNI=NR; AC=NR; AP=NR | eculizumab; index=Eculizumab only | Improved/recovered | Improved | Partial or mixed outcome; hematologic response; follow-up 9 months; remission reported |
| 42 | P043 | Kello 2019; case 1 | full_text | United States | F; 27.0 y | Class IV / diffuse proliferative LN | MAHA=present; PLT=present; AKI=present; biopsy-TMA=absent; RRT=absent | none clearly reported | not measured/not reported | T1=Y; strict T2=not documented; T3=not documented; VUS/uncertain=not documented; negative assay=not documented | PLEX=NR; CYC=Y; MMF=NR; RTX=NR; IVIG=NR; CNI=NR; AC=NR; AP=NR | eculizumab; index=Eculizumab only | Improved/recovered | Improved | Improved/recovered or dialysis-independent; hematologic response; follow-up 24 months |
| 43 | P044 | Kello 2019; case 2 | full_text | United States | F; 40.0 y | Unclear or non-LN dominant | MAHA=present; PLT=present; AKI=absent; biopsy-TMA=not_reported; RRT=not_reported | APS/CAPS/aPL | not measured/not reported | T1=Y; strict T2=not documented; T3=not documented; VUS/uncertain=not documented; negative assay=not documented | PLEX=NR; CYC=NR; MMF=NR; RTX=NR; IVIG=NR; CNI=NR; AC=Y; AP=NR | eculizumab; index=Eculizumab only | Death/no renal recovery | Improved | Partial or mixed outcome; hematologic response; death reported |
| 44 | P045 | Kello 2019; case 3 | full_text | United States | M; 57.0 y | Unclear or non-LN dominant | MAHA=present; PLT=present; AKI=present; biopsy-TMA=present; RRT=present | APS/CAPS/aPL | 0.59 | T1=not documented; strict T2=not documented; T3=not documented; VUS/uncertain=not documented; negative assay=not documented | PLEX=Y; CYC=NR; MMF=NR; RTX=NR; IVIG=NR; CNI=NR; AC=Y; AP=NR | eculizumab; index=Eculizumab only | Kidney function loss | Improved | Improved/recovered or dialysis-independent; hematologic response; survived |
| 45 | P046 | Kello 2019; case 4 | full_text | United States | F; 32.0 y | Unclear or non-LN dominant | MAHA=present; PLT=present; AKI=present; biopsy-TMA=present; RRT=present | APS/CAPS/aPL | 0.54 | T1=Y; strict T2=not documented; T3=not documented; VUS/uncertain=not documented; negative assay=not documented | PLEX=Y; CYC=NR; MMF=NR; RTX=NR; IVIG=NR; CNI=NR; AC=Y; AP=NR | eculizumab; index=Eculizumab only | Death/no renal recovery | Improved | Improved/recovered or dialysis-independent; hematologic response; death reported |
| 46 | P047 | Kello 2019; case 5 | full_text | United States | M; 27.0 y | Mixed proliferative + membranous LN | MAHA=present; PLT=present; AKI=present; biopsy-TMA=present; RRT=absent | APS/CAPS/aPL | 0.58 | T1=Y; strict T2=not documented; T3=not documented; VUS/uncertain=not documented; negative assay=not documented | PLEX=Y; CYC=NR; MMF=Y; RTX=NR; IVIG=NR; CNI=NR; AC=NR; AP=NR | eculizumab; index=Eculizumab only | Improved/recovered | Improved | Improved/recovered or dialysis-independent; hematologic response; follow-up 24 months |
| 47 | P048 | Kello 2019; case 6 | full_text | United States | F; 24.0 y | Class IV / diffuse proliferative LN | MAHA=present; PLT=present; AKI=present; biopsy-TMA=present; RRT=absent | APS/CAPS/aPL | 0.56 | T1=Y; strict T2=not documented; T3=not documented; VUS/uncertain=not documented; negative assay=not documented | PLEX=Y; CYC=Y; MMF=NR; RTX=NR; IVIG=NR; CNI=NR; AC=Y; AP=NR | eculizumab; index=Eculizumab only | Improved/recovered | Improved | Improved/recovered or dialysis-independent; hematologic response; follow-up 4 months |
| 48 | P049 | Kello 2019; case 7 | full_text | United States | F; 59.0 y | Unclear or non-LN dominant | MAHA=present; PLT=present; AKI=absent; biopsy-TMA=not_reported; RRT=not_reported | APS/CAPS/aPL | not measured/not reported | T1=not documented; strict T2=not documented; T3=not documented; VUS/uncertain=not documented; negative assay=not documented | PLEX=Y; CYC=Y; MMF=NR; RTX=Y; IVIG=NR; CNI=NR; AC=Y; AP=NR | eculizumab; index=Eculizumab only | Unclear | Improved | Partial or mixed outcome; hematologic response; survived |
| 49 | P050 | Kello 2019; case 8 | full_text | United States | M; 21.0 y | Class IV / diffuse proliferative LN | MAHA=present; PLT=present; AKI=present; biopsy-TMA=present; RRT=absent | none clearly reported | 0.69 | T1=Y; strict T2=not documented; T3=not documented; VUS/uncertain=not documented; negative assay=not documented | PLEX=Y; CYC=NR; MMF=NR; RTX=Y; IVIG=NR; CNI=NR; AC=NR; AP=NR | eculizumab; index=Eculizumab only | Improved/recovered | Improved | Improved/recovered or dialysis-independent; hematologic response; follow-up 1 months |
| 50 | P051 | Kello 2019; case 9 | full_text | United States | M; 21.0 y | Mixed proliferative + membranous LN | MAHA=present; PLT=present; AKI=present; biopsy-TMA=absent; RRT=present | none clearly reported | 0.67 | T1=Y; strict T2=not documented; T3=not documented; VUS/uncertain=not documented; negative assay=not documented | PLEX=Y; CYC=Y; MMF=NR; RTX=NR; IVIG=NR; CNI=NR; AC=NR; AP=NR | eculizumab; index=Eculizumab only | Kidney function loss | Improved | Improved/recovered or dialysis-independent; hematologic response; follow-up 1 months |
| 51 | P052 | Skoczyń-ska 2019; case 1 | published_abstract | Poland | F; 35.0 y | LN, class not specified | MAHA=not_reported; PLT=present; AKI=present; biopsy-TMA=not_reported; RRT=present | APS/CAPS/aPL; infection | NR | T1=Y; strict T2=not documented; T3=not documented; VUS/uncertain=not documented; negative assay=not documented | PLEX=Y; CYC=NR; MMF=NR; RTX=NR; IVIG=NR; CNI=NR; AC=Y; AP=NR | eculizumab; index=Eculizumab only | Kidney function loss | Unclear | Kidney function loss / dialysis-dependent; hematologic response |
| 52 | P053 | Drexler 2021; case 1 | published_abstract | United States | F; 45.0 y | LN, class not specified | MAHA=present; PLT=present; AKI=present; biopsy-TMA=not_reported; RRT=present | none clearly reported | 0.18 | T1=Y; strict T2=not documented; T3=not documented; VUS/uncertain=not documented; negative assay=not documented | PLEX=NR; CYC=NR; MMF=NR; RTX=NR; IVIG=Y; CNI=NR; AC=NR; AP=NR | eculizumab; index=Eculizumab only | Death/no renal recovery | Improved | Unclear/not reported; hematologic response; death reported |
| 53 | P054 | Kim 2021; case 1 | full_text | South Korea | M; 23.0 y | Class IV / diffuse proliferative LN | MAHA=present; PLT=present; AKI=present; biopsy-TMA=present; RRT=present | none clearly reported | 0.9 | T1=Y; strict T2=not documented; T3=not documented; VUS/uncertain=not documented; negative assay=not documented | PLEX=Y; CYC=Y; MMF=NR; RTX=NR; IVIG=NR; CNI=NR; AC=NR; AP=NR | eculizumab; index=Eculizumab only | Improved/recovered | Improved | Improved/recovered or dialysis-independent; hematologic response; remission reported |
| 54 | P055 | Su 2021; case 1 | full_text | China | F; 56.0 y | LN, class not specified | MAHA=present; PLT=present; AKI=present; biopsy-TMA=absent; RRT=not_reported | APS/CAPS/aPL | 0.848 | T1=Y; strict T2=not documented; T3=not documented; VUS/uncertain=not documented; negative assay=Y | PLEX=Y; CYC=NR; MMF=Y; RTX=Y; IVIG=Y; CNI=NR; AC=Y; AP=NR | none reported; index=No complement inhibitor | Improved/recovered | No response | Improved/recovered or dialysis-independent; no hematologic recovery; survived |
| 55 | P056 | Su 2021; case 2 | full_text | China | F; 58.0 y | LN, class not specified | MAHA=unclear; PLT=unclear; AKI=unclear; biopsy-TMA=not_reported; RRT=present | none clearly reported | normal | T1=not documented; strict T2=not documented; T3=not documented; VUS/uncertain=Y; negative assay=not documented | PLEX=Y; CYC=NR; MMF=NR; RTX=NR; IVIG=Y; CNI=NR; AC=NR; AP=NR | none reported; index=No complement inhibitor | Death/no renal recovery | No response | Kidney function loss / dialysis-dependent; hematologic response; death reported |
| 56 | P057 | Torres 2021; case 1 | full_text | United States | F; 23.0 y | Class IV / diffuse proliferative LN | MAHA=present; PLT=present; AKI=present; biopsy-TMA=present; RRT=present | pregnancy/postpartum | 0.62 | T1=Y; strict T2=Y; T3=not documented; VUS/uncertain=not documented; negative assay=Y | PLEX=Y; CYC=Y; MMF=Y; RTX=NR; IVIG=No; CNI=NR; AC=NR; AP=NR | eculizumab; index=Eculizumab only | Kidney function loss | Improved | Improved/recovered or dialysis-independent; no recurrence reported |
| 57 | P058 | Xie 2021; case 1 | published_abstract | United States | M; 15.0 y | Class IV / diffuse proliferative LN | MAHA=unclear; PLT=present; AKI=present; biopsy-TMA=not_reported; RRT=present | none clearly reported | NR | T1=not documented; strict T2=not documented; T3=not documented; VUS/uncertain=Y; negative assay=not documented | PLEX=NR; CYC=NR; MMF=Y; RTX=NR; IVIG=NR; CNI=NR; AC=NR; AP=NR | eculizumab; index=Eculizumab only | Kidney function loss | Unclear | Kidney function loss / dialysis-dependent; hematologic response |
| 58 | P059 | AlGhobaishi 2022; case 1 | full_text | Saudi Arabia | F; 13.0 y | LN, class not specified | MAHA=present; PLT=present; AKI=present; biopsy-TMA=present; RRT=present | neurologic involvement | severe deficiency 0.39 IU/dL | T1=Y; strict T2=not documented; T3=not documented; VUS/uncertain=not documented; negative assay=not documented | PLEX=Y; CYC=Y; MMF=Y; RTX=Y; IVIG=NR; CNI=NR; AC=NR; AP=NR | eculizumab; index=Eculizumab only | Improved/recovered | Improved | Improved/recovered or dialysis-independent; short-term follow-up |
| 59 | P060 | Alhamoud 2022; case 1 | full_text | United States | M; 18.0 y | Class IV / diffuse proliferative LN | MAHA=present; PLT=present; AKI=present; biopsy-TMA=present; RRT=not_reported | APS/CAPS/aPL; malignant/severe hypertension; neurologic involvement | normal | T1=Y; strict T2=not documented; T3=not documented; VUS/uncertain=not documented; negative assay=Y | PLEX=Y; CYC=Y; MMF=Y; RTX=NR; IVIG=NR; CNI=NR; AC=NR; AP=NR | eculizumab; index=Eculizumab only | Improved/recovered | Improved | Improved/recovered or dialysis-independent; hematologic response; follow-up 12 months; no recurrence reported |
| 60 | P061 | Amin 2023; case 1 | published_abstract | United States | F; 21.0 y | Class IV / diffuse proliferative LN | MAHA=present; PLT=present; AKI=present; biopsy-TMA=present; RRT=present | APS/CAPS/aPL; neurologic involvement | normal | T1=Y; strict T2=not documented; T3=not documented; VUS/uncertain=Y; negative assay=not documented | PLEX=Y; CYC=NR; MMF=Y; RTX=NR; IVIG=NR; CNI=NR; AC=NR; AP=NR | ravulizumab; index=Other complement-inhibitor sequence | Improved/recovered | Improved | Improved/recovered or dialysis-independent; hematologic response |
| 61 | P062 | Brill 2023; case 1 | published_abstract | United States | F; 21.0 y | Unclear or non-LN dominant | MAHA=present; PLT=present; AKI=present; biopsy-TMA=not_reported; RRT=present | DAH/lung involvement; neurologic involvement | normal | T1=not documented; strict T2=not documented; T3=Y; VUS/uncertain=Y; negative assay=Y | PLEX=Y; CYC=Y; MMF=NR; RTX=Y; IVIG=NR; CNI=NR; AC=NR; AP=NR | ravulizumab; index=Other complement-inhibitor sequence | Kidney function loss | Improved | Kidney function loss / dialysis-dependent; hematologic response; discharged |
| 62 | P063 | Lin 2023; case 1 | published_abstract | United States | M; 19.0 y | Class IV / diffuse proliferative LN | MAHA=present; PLT=present; AKI=present; biopsy-TMA=unclear; RRT=present | none clearly reported | 0.58 | T1=not documented; strict T2=not documented; T3=not documented; VUS/uncertain=not documented; negative assay=not documented | PLEX=Y; CYC=NR; MMF=Y; RTX=NR; IVIG=NR; CNI=NR; AC=NR; AP=NR | eculizumab; index=Eculizumab only | Improved/recovered | Improved | Improved/recovered or dialysis-independent; hematologic response; discharged |
| 63 | P064 | Díaz Góngora 2024; case 1 | published_abstract | Argentina | F; 18.0 y | Class IV / diffuse proliferative LN | MAHA=absent; PLT=not_reported; AKI=not_reported; biopsy-TMA=absent; RRT=present | none clearly reported | NR | T1=Y; strict T2=not documented; T3=not documented; VUS/uncertain=not documented; negative assay=Y | PLEX=Y; CYC=Y; MMF=Y; RTX=Y; IVIG=NR; CNI=NR; AC=NR; AP=NR | eculizumab; index=Eculizumab only | Kidney function loss | Unclear | Kidney function loss / dialysis-dependent; hematologic response |
| 64 | P065 | Smith 2024; case 1 | full_text | Canada | F; 23.0 y | Class IV / diffuse proliferative LN | MAHA=present; PLT=present; AKI=present; biopsy-TMA=present; RRT=absent | none clearly reported | low-normal (0.12 U/mL); inhibitor negative | T1=Y; strict T2=not documented; T3=not documented; VUS/uncertain=Y; negative assay=Y | PLEX=Y; CYC=No; MMF=Y; RTX=No; IVIG=NR; CNI=NR; AC=NR; AP=NR | eculizumab; index=Eculizumab only | Improved/recovered | Improved | Improved/recovered or dialysis-independent; hematologic response; no recurrence reported |
| 65 | P066 | Benvenuti 2025; case 1 | full_text | Italy | M; 59.0 y | LN, class not specified | MAHA=present; PLT=present; AKI=present; biopsy-TMA=absent; RRT=present | none clearly reported | 0.4 | T1=Y; strict T2=not documented; T3=not documented; VUS/uncertain=not documented; negative assay=Y | PLEX=Y; CYC=NR; MMF=Y; RTX=Y; IVIG=NR; CNI=NR; AC=NR; AP=NR | eculizumab, iptacopan; index=Other complement-inhibitor sequence | Kidney function loss | Improved | Kidney function loss / dialysis-dependent; hematologic response |
| 66 | P067 | Inatomi 2025; case 1 | full_text | Japan | F; 33.0 y | LN, class not specified | MAHA=present; PLT=present; AKI=present; biopsy-TMA=not_reported; RRT=present | pregnancy/postpartum; DAH/lung involvement | 60%, inhibitor negative | T1=not documented; strict T2=not documented; T3=not documented; VUS/uncertain=not documented; negative assay=Y | PLEX=No; CYC=NR; MMF=NR; RTX=NR; IVIG=NR; CNI=Y; AC=NR; AP=No | none reported; index=No complement inhibitor | Improved/recovered | Improved | Improved/recovered or dialysis-independent; hematologic response; discharged |
| 67 | P068 | Thomson 2025; case 1 | full_text | Australia | F; NR y | Class IV / diffuse proliferative LN | MAHA=present; PLT=present; AKI=present; biopsy-TMA=absent; RRT=present | infection | 31.9%, within normal range | T1=Y; strict T2=not documented; T3=not documented; VUS/uncertain=not documented; negative assay=not documented | PLEX=NR; CYC=Y; MMF=Y; RTX=Y; IVIG=NR; CNI=Y; AC=NR; AP=NR | eculizumab; index=Eculizumab only | Improved/recovered | Improved | Partial or mixed outcome; hematologic response; follow-up 2 years; remission reported |
| 68 | P069 | Yu 2026; case 1 | full_text | China | M; 20.0 y | Mixed proliferative + membranous LN | MAHA=present; PLT=present; AKI=present; biopsy-TMA=not_reported; RRT=present | none clearly reported | 26.61%, inhibitor negative; not severe deficiency | T1=Y; strict T2=not documented; T3=Y; VUS/uncertain=not documented; negative assay=not documented | PLEX=NR; CYC=NR; MMF=Y; RTX=Y; IVIG=Y; CNI=Y; AC=NR; AP=NR | eculizumab; index=Eculizumab only | Improved/recovered | Improved | Improved/recovered or dialysis-independent; hematologic response; remission reported; dialysis-independent |
| 69 | P070 | Sun 2026; patient 5 | full_text | China | F; 22.0 y | LN III+V; renal-biopsy TMA; AI4 CI8; chronic tubulointerstitial damage | MAHA=not_reported; PLT=not_reported; AKI=unclear; biopsy-TMA=present; RRT=present | APS/pregnancy/HTN/infection/drug adjudication NR | NR | T1=not documented; strict T2=not documented; T3=not documented; VUS/uncertain=not documented; negative assay=not documented | PLEX=NR; CYC=NR; MMF=Y; RTX=NR; IVIG=NR; CNI=NR; AC=NR; AP=NR | none reported; index=No complement inhibitor | Kidney function loss | Unclear | 3 months |
| 70 | P071 | Pokharel 2026; case 1 | full_text | USA | F; 36.0 y | Known SLE; class III LN; prominent biopsy TMA; collapsing glomerulopathy; AI3/24 CI8/12 | MAHA=present; PLT=present; AKI=present; biopsy-TMA=present; RRT=present | Severe HTN ~190; APS negative; no causative drug | Not severely reduced; reported elevated/>10%, no exact value | T1=Y; strict T2=not documented; T3=Y; VUS/uncertain=not documented; negative assay=not documented | PLEX=Y; CYC=NR; MMF=Y; RTX=NR; IVIG=Y; CNI=NR; AC=NR; AP=NR | eculizumab; index=Eculizumab only | Kidney function loss | Unclear | 12 days to transfer |
| 71 | P072 | Lin 2026; case 1 | full_text | China | M; 49.0 y | New SLE; proteinuria 567 mg/24h; no kidney biopsy/LN class | MAHA=present; PLT=present; AKI=absent; biopsy-TMA=not_reported; RRT=not_reported | DAT IgG2+ immune-hemolysis overlap; aPL negative; no other trigger reported | 75.41%; inhibitor negative | T1=Y; strict T2=not documented; T3=Y; VUS/uncertain=not documented; negative assay=not documented | PLEX=No; CYC=Y; MMF=NR; RTX=NR; IVIG=NR; CNI=NR; AC=NR; AP=NR | eculizumab; index=Eculizumab only | Improved/recovered | Improved | 6 days after eculizumab/discharge |
| 72 | P073 | Nickavar 2026; case 1 | full_text | Iran | F; 13.0 y | New SLE; LN IV-G(A/C); glomerular/arteriolar TMA; AI7/24 CI2-3/12 | MAHA=present; PLT=present; AKI=present; biopsy-TMA=present; RRT=not_reported | HTN160/90; LA/aCL/beta2GP1 positive; APS overlap; no pregnancy/infection/drug | Normal | T1=Y; strict T2=not documented; T3=not documented; VUS/uncertain=not documented; negative assay=Y | PLEX=Y; CYC=Y; MMF=NR; RTX=Y; IVIG=Y; CNI=NR; AC=NR; AP=Y | eculizumab; index=Eculizumab only | Improved/recovered | Improved | 2 years |
| 73 | P074 | Stirnkorb 2026; case 1 | published_abstract | Germany | F; 12.0 y | Severe SLE/LN and refractory TMA; class/pathology NR in abstract | MAHA=present; PLT=not_reported; AKI=unclear; biopsy-TMA=not_reported; RRT=present | APS/pregnancy/HTN/infection/drug NR | NR | T1=not documented; strict T2=not documented; T3=not documented; VUS/uncertain=not documented; negative assay=not documented | PLEX=NR; CYC=NR; MMF=NR; RTX=NR; IVIG=NR; CNI=NR; AC=NR; AP=NR | eculizumab, pegcetacoplan; index=Other complement-inhibitor sequence | Improved/recovered | Improved | Post-HD withdrawal follow-up NR |
| 74 | P075 | Fukunaga 2026; case 1 | full_text | Japan | F; 69.0 y | SLE and biopsy-diagnosed LN since age27; class NR; no repeat biopsy | MAHA=present; PLT=present; AKI=present; biopsy-TMA=not_reported; RRT=not_reported | Active SLE SLEDAI7; tacrolimus; Klebsiella pyelonephritis/bacteremia; later CMV/recurrent UTI; etiology multifactorial | 16%; inhibitor negative | T1=Y; strict T2=not documented; T3=not documented; VUS/uncertain=not documented; negative assay=not documented | PLEX=Y; CYC=NR; MMF=Y; RTX=NR; IVIG=NR; CNI=Y; AC=NR; AP=NR | none reported; index=No complement inhibitor | Improved/recovered | Improved | 89 days/no post-discharge follow-up |
| 75 | P076 | Baltaci 2026; case 1 | full_text | Turkey | F; 30.0 y | New SLE; LN IV+V; extensive glomerular/arteriolar fibrin thrombi | MAHA=present; PLT=present; AKI=unclear; biopsy-TMA=present; RRT=not_reported | Purtscher-like retinopathy; tamponade; DAH; DAT+; all aPL negative; no other trigger | 37.5% | T1=Y; strict T2=not documented; T3=not documented; VUS/uncertain=not documented; negative assay=not documented | PLEX=Y; CYC=Y; MMF=Y; RTX=NR; IVIG=Y; CNI=NR; AC=NR; AP=NR | eculizumab; index=Eculizumab only | Improved/recovered | Improved | 4-6 months; continuing follow-up |
| 76 | P077 | Kaneda 2020; case 1 | full_text | Japan | F; 5.0 y | New SLE; LN IV-S(A); mesangiolysis/endocapillary proliferation/endothelial swelling/fibrin; biopsy TMA | MAHA=present; PLT=present; AKI=unclear; biopsy-TMA=present; RRT=not_reported | Severe HTN; recent diarrhea but stool culture negative; aPL/Coombs negative; no drug trigger | Normal | T1=Y; strict T2=not documented; T3=not documented; VUS/uncertain=not documented; negative assay=Y | PLEX=Y; CYC=Y; MMF=Y; RTX=NR; IVIG=NR; CNI=Y; AC=Y; AP=NR | eculizumab; index=Eculizumab only | Improved/recovered | Improved | ~31 months |
| 77 | P078 | Khan 2020; case 1 | published_abstract | USA | M; 29.0 y | SLE diagnosed 3 months earlier; class IV-S(A/C)+V lupus nephritis with renal-biopsy TMA | MAHA=present; PLT=present; AKI=present; biopsy-TMA=present; RRT=present | Diffuse alveolar hemorrhage; infection was reported in the abstract extraction | Normal | T1=Y; strict T2=not documented; T3=not documented; VUS/uncertain=not documented; negative assay=not documented | PLEX=Y; CYC=Y; MMF=NR; RTX=NR; IVIG=NR; CNI=NR; AC=NR; AP=NR | eculizumab; index=Eculizumab only | Unclear | Unclear | Transferred to ward by day 10, then left hospital; no longer-term follow-up |
| 78 | P079 | Theodoru 2023; case 1 | published_abstract | USA | M; 43.0 y | SLE; class III lupus nephritis; renal-biopsy TMA detail not reported in abstract | MAHA=present; PLT=present; AKI=present; biopsy-TMA=not_reported; RRT=not_reported | Cat-bite infection; Graves disease; hypertension; diffuse alveolar hemorrhage | Not reported | T1=not documented; strict T2=not documented; T3=not documented; VUS/uncertain=Y; negative assay=not documented | PLEX=Y; CYC=NR; MMF=NR; RTX=Y; IVIG=NR; CNI=NR; AC=NR; AP=NR | eculizumab; index=Eculizumab only | Unclear | Unclear | Discharge to rehabilitation; no longer-term follow-up reported |
